# Understanding psychiatrist readiness for AI: a study of access, self-efficacy, trust, and design expectations

**DOI:** 10.1186/s12913-026-14010-6

**Published:** 2026-01-17

**Authors:** Yue He, Francis Xiatian Zhang, Xiaxia Wu, Meng Fang, Sisi Zheng, Hong Zhu

**Affiliations:** 1 Beijing Key Laboratory of Mental Disorders, Beijing Anding Hospital, National Clinical Research Center for Mental Disorders & National Center for Mental Disorders, Capital Medical University, Beijing, 100088 China; 2 Institute for Regeneration and Repair, University of Edinburgh, Edinburgh, EH16 4TJ UK; 3 School of Chinese Materia Medica, Beijing University of Chinese Medicine, Beijing, 100029 China; 4 Beijing Tongrentang Chinese Medicine Formula Granules Investment Limited Company, Beijing, 100061 China

**Keywords:** AI, Psychiatrist, Readiness, Access, Self-efficacy, Trust, Design expectations

## Abstract

**Background:**

Artificial intelligence (AI) is receiving growing attention in psychiatric practice, yet psychiatrists vary considerably in how they perceive its benefits, risks, and clinical usefulness. Successful implementation depends not only on technological performance but also on clinicians’ readiness, including their access to AI, confidence in using it, trust in its reliability, and expectations for its design. Evidence on these dimensions remains limited in China.

**Objective:**

This study examined Chinese psychiatrists’ readiness for AI across four dimensions—access, self-efficacy, trust, and design expectations—and explored variation across demographic and professional subgroups.

**Method:**

A cross-sectional online survey was distributed through the WeChat platform from March 20 to 22, 2025. Eligible participants were licensed psychiatrists engaged in clinical practice. A total of 134 valid responses were obtained from clinicians across diverse provinces, hospital tiers, and professional roles. Descriptive analyses and group comparisons were conducted.

**Results:**

Psychiatrists reported broad exposure to AI, though knowledge was largely acquired through informal rather than structured sources. Overall self-efficacy was moderate, with higher confidence observed among younger clinicians, male clinicians, and those who had received AI-related training. Trust in AI was generally positive, and department heads expressed especially strong confidence in its future role. Across subgroups, psychiatrists consistently prioritized AI applications that reduce administrative and documentation burden, while expressing lower expectations for AI use in communication, assessment, or psychotherapy.

**Conclusion:**

Chinese psychiatrists demonstrated cautious optimism and moderate readiness for AI. Limited access to formal training and subgroup differences in confidence highlight the need for targeted capacity-building. The strong preference for documentation-support tools underscores the importance of designing AI systems that integrate smoothly into clinical workflows while preserving the human-centered nature of psychiatric care.

**Supplementary Information:**

The online version contains supplementary material available at 10.1186/s12913-026-14010-6.

## Background

Artificial intelligence (AI) has shown growing potential in clinical psychiatry [1]. In this study, “AI” primarily refers to language-based models (e.g., ChatGPT, Bard) and decision-support systems used for documentation, diagnosis, and patient communication. With advances in large language models (LLMs), these tools are increasingly incorporated into clinical practice and research [2–4]. With rising global mental-health needs and increasing pressure on service capacity [5], psychiatry has emerged as a priority area for AI-enabled support, where current LLM applications adapted from general medical models offer functions such as symptom identification, treatment suggestions, and triage support [6]. MedGPT, for instance, assists with history-taking and treatment planning [7], though its psychiatric use remains limited [8, 9]. Other models, including ClinicalBERT and BioBERT, support literature mining and text-based symptom recognition but cannot process multimodal psychiatric information (e.g., speech) [10, 11], and still provide simplified reasoning for complex conditions [12].

In China, AI use in psychiatry is early but expanding. Diagnostic innovations include GAN-based fMRI classification of schizophrenia and depression [13], metabolomic biomarkers for depression [14], and predictive analytics for postoperative delirium [15]. Beyond diagnosis, AI has been explored for crisis-intervention hotlines [16] and psychiatric rehabilitation outcome prediction [17]. These developments suggest a growing ecosystem of AI-related work in Chinese psychiatry, but they focus predominantly on technical performance and clinical outcomes rather than the preparedness of psychiatrists themselves to engage with AI. This mismatch highlights a critical gap in the Chinese context, where rapid technological development may outpace clinicians’ readiness.

Recent studies show that many psychiatrists express openness toward AI adoption [18], frequently valuing its potential to reduce documentation burden and improve access, consistent with findings that AI can support assessment and psychotherapy delivery [19, 20]. Others remain cautious, citing risks such as missed nuanced information, model opacity [21], underperformance in complex scenarios [22], or misjudgment of suicide risk [23]. Concerns about overreliance, workflow disruption, privacy, medico-legal issues, and stigma further contribute to hesitation [24]. Broader challenges—data security, algorithmic bias, and reliability [25, 26] amid evolving regulations [27]—intensify ambivalence regarding AI’s maturity and safety in psychiatry.

Despite AI’s potential, adoption across healthcare—psychiatry included—has been uneven and slower than anticipated [28]. Evidence on how best to integrate AI into psychiatric workflows remains limited. Existing studies often focus on technical performance rather than clinicians’ perspectives or practical needs, and physician-feedback research on specific AI tools [29–31] lacks generalizability and rarely examines determinants of real-world implementation. This gap risks misalignment between AI development priorities and clinical realities, underscoring the importance of understanding how psychiatrists perceive and interact with AI, and what conditions enable or hinder their day-to-day use. These limitations highlight that effective AI integration depends not only on the technology itself but on clinicians’ readiness to engage with it.

Professionals’ “readiness” is a key precursor to successful adoption. Rather than attitudes alone, readiness encompasses systemic, cognitive, and practical capacities. Evidence suggests readiness frameworks must incorporate trust, facilitating conditions, and appropriate training [32]. We conceptualize psychiatrists’ AI readiness as shaped by four main determinants: Access (organizational and technical support) [33], Self-Efficacy (confidence in using AI) [34], Trust (expectations related to robustness, explainability, privacy, and fairness) [35], and Design Expectations (usability and support for relational and shared-decision-making processes) [36]. This framework links concrete, measurable dimensions to the broader readiness construct and provides a basis for empirical assessment.

As key stakeholders, physicians play a central role in shaping and adopting AI technologies [37]. Psychiatrists obtain AI knowledge through diverse channels, resulting in differences in confidence, trust, and expectations across clinical roles [38]. For example, residents primarily handle documentation and routine assessments [39], where natural language processing tools could reduce administrative burden; attending psychiatrists focus on diagnosis, treatment, and risk evaluation, where machine learning may support decision-making; and department heads or senior leaders manage complex cases and strategic planning, making them well positioned to use AI for data-driven research and organizational innovation [40]. Effective psychiatric AI systems should therefore offer adaptable, role-specific functionalities that match the varied needs of clinical practice.

In summary, this study addresses the above gaps by examining four determinants of psychiatrists’ readiness for AI—access, self-efficacy, trust, and design expectations—within the context of Chinese psychiatric practice. Using a cross-sectional survey of psychiatrists from diverse healthcare institutions across China, we (1) describe their current levels of AI access, self-efficacy, trust, and design expectations; (2) analyze how these readiness dimensions vary by gender, age, hospital tier, professional title, and AI training experience; and (3) identify which AI application scenarios psychiatrists prioritize in clinical work. These objectives aim to guide the design and implementation of AI systems aligned with psychiatric workflows and clinicians’ needs.

## Methods

### Research participants

On March 20, 2025, an online questionnaire was distributed for this cross-sectional study on the WeChat platform (a widely used online social platform in China), with practicing psychiatrists selected as the research participants. Inclusion criteria were as follows: (1) holding a valid license as a psychiatrist; (2) currently engaged in clinical psychiatric practice; (3) providing informed consent. Individuals no longer working in clinical settings or with incomplete questionnaires were excluded.

This study employed a questionnaire survey method, with the questionnaire comprising 10 core items. The sample size was determined based on widely accepted empirical guidelines for factor analysis reliability, which recommend that the effective sample size should be 5 to 10 times the number of questionnaire items. Accordingly, the minimum required sample size for this study was 50 to 100 responses. Considering potential invalid responses and aiming to enhance the stability and rigorousness of statistical results, the target sample size was set at more than 130 responses.

The questionnaire was collected on March 22, 2025. These psychiatrists were affiliated with mental health care facilities or general hospitals with psychiatric departments across various provinces and cities in China, representing a range of institutional tiers. All participants took part voluntarily. A total of 134 valid responses were collected and included in the final analysis.

### Survey instrument

The questionnaire consists of four major components, covering demographic information, access, self-efficacy and trust, and design expectations. First, it collected demographic information, including gender, age, education level, clinical role, job title, and hospital tier. Second, access refers to the way and habits psychiatrists engage with AI. It assessed AI knowledge sources through a multiple-choice question covering nine possible channels (e.g., professional journals, training programs, social media, peer discussions), a 5-point Likert scale evaluating frequency of reading AI-related content, exposure to AI at work, and self-directed learning, as well as a yes/no item on whether the respondent had participated in AI-related training, followed by a rating of its helpfulness. Third, Self-efficacy refers to psychiatrists’ personal assessment of their ability to effectively utilize AI, while trust reflects their evaluation of the AI system itself. AI self-efficacy and trust were measured using 5-point scales assessing confidence in understanding AI, perceived ability to use AI effectively, and trust in AI’s future clinical role. Fourth, design expectations refer to psychiatrists’ anticipated directions for the future design and optimization of AI. design expectations were assessed by asking respondents to rank the importance, demand, potential, expectations of AI across three functional domains (clinical care, science communication, and administrative management) and nine medical scenarios (patient management, medical history collection, psychiatric examination, medical documentation, diagnostic assistance, treatment planning and efficacy prediction, risk assessment and prognosis estimation, doctor-patient communication, and psychological intervention).

### Statistical analysis

Data was analyzed using R software. Because the online survey platform required all items to be completed before submission, the final dataset contained no missing values, and no additional data-handling procedures were necessary. Categorical variables are presented as frequencies and percentages (n, %), normally distributed continuous variables as means and standard deviations (𝑥̅ ± 𝑠), and ordinal or non-normally distributed variables as medians, interquartile ranges (IQR), and modes. The assumptions for statistical tests were rigorously checked. Specifically, the normality of data distribution was assessed to determine the appropriateness of using parametric (t-test/ANOVA) versus non-parametric tests. Internal consistency reliability was assessed using Cronbach’s alpha coefficient. Construct validity was examined through Kaiser-Meyer-Olkin (KMO) measure of sampling adequacy, Bartlett’s test of sphericity, and exploratory factor analysis (EFA) using maximum likelihood extraction with oblimin rotation for multi-factor solutions. Five scales were examined: ① AI Access Scale (Q9): 3 items measuring exposure to AI technology and proactive learning behaviors; ② AI Self-Efficacy Scale (Q10): 3 items assessing understanding of AI and expectations for its role; ③ Medical Demand Scale (Q15): 9 items assessing perceived needs for AI in medical practice; ④ Medical Potential Scale (Q16): 9 items evaluating the perceived potential of AI applications; ⑤ Medical Design Expectations Scale (Q17): 9 items measuring willingness to adopt AI tools.

Descriptive statistics were used to summarize sources of AI knowledge, level of AI understanding, and perceived importance, demand, and potential of AI across medical scenarios. To explore group differences, non-parametric omnibus tests were first used to assess overall differences across groups. When significant, post-hoc pairwise comparisons were conducted with False Discovery Rate (FDR) correction, with the significance level set at α = 0.05, to appropriately identify specific group differences while controlling multiple testing in ordinal survey data. As this study was primarily descriptive and exploratory, aiming to characterize group differences rather than build explanatory models, we did not perform multivariable adjustments or calculate formal effect-size indices. Instead, we focused on transparent reporting of test statistics, p-values, and FDR-adjusted significance levels.

### Research findings

This section presents the survey results, including participant demographics, access to AI knowledge, self-efficacy and trust in AI, perceived needs, and expectations for AI in psychiatric practice, along with related influencing factors.

### Demographic characteristics

A total of 139 questionnaires were distributed in this study, and 135 were returned, among which 134 were valid. One was excluded because the respondent’s position was not a doctor, resulting in an effective return rate of 96.40%. Demographic characteristics of the 134 respondents are summarized in Table 1.

**Table 1:** Participant demographics

project	frequency	percentage
gender		
man	48	35.80
woman	86	64.20
age		
18–23 years old	1	0.70
24–30 years old	45	33.60
31–40 years old	55	41.00
41–50 years old	28	20.90
Over 50 years old	5	3.70
Degree		
Specialist	3	2
undergraduate	51	38.10
Master	62	46.30
doctor	18	13.40
office		
Department head	15	11.20
Non-managerial positions	119	88.80
Instructors	17	12.60
Non-instructor	118	87.40
Scientific research posts	10	7.50
Non-scientific research posts	124	92.50
job title		
Junior physicians	42	31
Attending	37	27.60
Deputy Chief Physician	27	20.10
Chief physician	13	9.70
Reluctance to disclose	15	11.20

### Psychometric properties

Table 2 provides a comprehensive summary of psychometric properties for all five scales.

**Table 2:** Comprehensive summary of psychometric properties

Scale	Items	α	KMO	Factors	RMSEA	TLI
AI Exposure & Learning	3	0.867	0.728	1		-Inf
AI trust & Expectations	3	0.761	0.639	1		
Medical Demand	9	0.889	0.851	3	0.000	1.006
Medical Potential	9	0.920	0.869	2	0.166	0.845
Medical Optimization	9	0.928	0.861	2	0.190	0.827

Note. *N* = 134. α = Cronbach’s alpha; KMO = Kaiser-Meyer-Olkin measure; RMSEA = Root Mean Square Error of Approximation; TLI = Tucker-Lewis Index

Exploratory Factor Analysis (EFA) was conducted to examine the underlying structure of the Medical Need, Potential, and Optimization scales. The extraction method used was Maximum Likelihood with Oblimin rotation to account for potential correlations between factors. The factor loadings, communalities (h²), and factor structures are summarized in Table 3.

For the Medical Need Scale, the analysis revealed a three-factor solution. Factor 1, labeled “Advanced Clinical Decision Support Needs,” comprised items related to complex clinical tasks such as diagnostic assistance, treatment planning, and risk assessment. Factor 2, labeled “Basic Clinical Administration Needs,” included items focused on routine workflows, including patient management, medical history collection, and documentation. Factor 3, labeled “Psychological & Interaction Support Needs,” consisted of items involving doctor-patient communication and psychological interventions, reflecting the interpersonal aspects of care.

Both the Medical Potential Scale and the Medical Optimization Scale exhibited a stable two-factor structure. Factor 1 in both scales was identified as “Core Clinical Efficiency” (Potential/Optimization), clustering items related to the efficiency of core medical tasks and administrative processes. Factor 2 was labeled “Empathy & Humanistic Care” (Potential/Optimization), capturing items related to mental status examination, communication, and psychological support, which require a higher degree of human empathy and interaction.

All items showed satisfactory communalities (h²), indicating that the extracted factors adequately explained the variance in the observed variables.

**Table 3:** Factors of scales

	Medical Needs Scale				Medical Potential Scale			Medical Design Expectations Scale		
**Item**	**F1**	**F2**	**F3**	**h²**	**F1**	**F2**	**h²**	**F1**	**F2**	**h²**
Patient Management	0.136	**0.569**	0.05	0.464	**0.498**	0.334	0.569	**0.774**	0.044	0.641
Medical History Collection	-0.002	**0.916**	-0.001	0.837	**0.654**	0.11	0.531	**0.567**	0.221	0.518
Mental Status Examination	0.005	**0.607**	0.318	0.654	0.407	**0.431**	0.572	0.407	**0.466**	0.607
Medical Documentation Writing	0.404	**0.42**	-0.054	0.501	**0.702**	-0.076	0.432	**0.843**	-0.145	0.587
Diagnostic Assistance	**0.681**	0.291	-0.091	0.715	**0.88**	-0.046	0.727	**0.754**	0.123	0.692
Treatment Planning and Outcome Prediction	**0.974**	-0.061	-0.014	0.876	**0.911**	-0.022	0.805	**0.932**	-0.012	0.854
Risk Assessment and Prognosis Estimation	**0.861**	-0.013	0.133	0.824	**0.912**	0.025	0.862	**0.921**	0.005	0.855
Doctor-Patient Communication	-0.015	0.102	**0.803**	0.721	-0.044	**0.977**	0.904	0.106	**0.854**	0.848
Psychological Interventions	0.056	-0.039	**0.861**	0.745	0.092	**0.733**	0.631	-0.066	**0.97**	0.87

Note. Extraction method: Maximum Likelihood. Rotation: Oblimin. h² = Communality. Medical Need Scale: F1, Advanced Clinical Decision Support Needs; F2, Basic Clinical Administration Needs; F3, Psychological & Interaction Support Needs; Medical Potential Scale: F1, Core Clinical Efficiency Potential; F2, Empathy & Humanistic Care Potential; Medical Optimization: F1, Core Clinical Efficiency Optimization; F2, Empathy & Humanistic Care Optimization

### Psychiatrists’ AI access

#### Overview of AI access

**Fig. 1:**
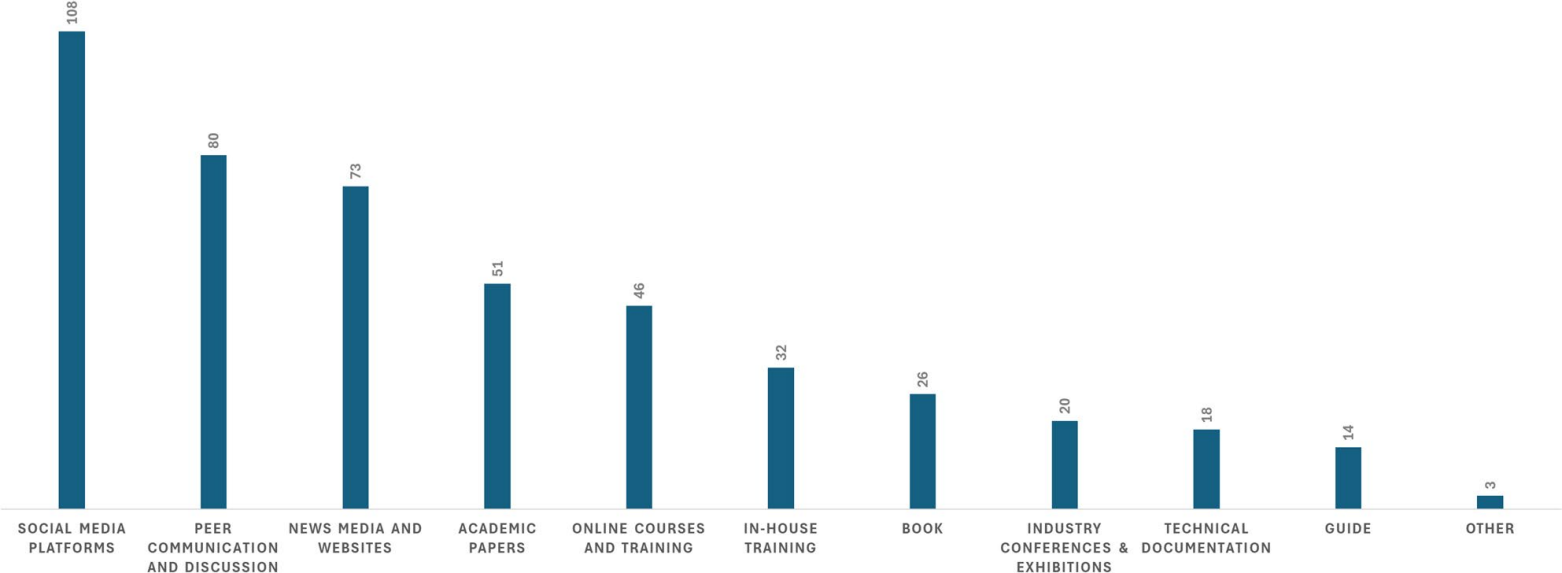
Reported sources of AI-related knowledge among psychiatrists (*N* = 134)

This subsection summarizes where psychiatrists most commonly acquire AI-related knowledge. The distribution of AI knowledge sources is illustrated in Fig. 1. The most common sources of AI knowledge were social media (108), peer discussions (80), and news media (73). Less frequent sources included academic papers, training programs, books, conferences, manufacturer documentation, and government reports (Fig. 1).

Psychiatrists rated their agreement with the statement regarding the acquisition of AI technology on a 1–5 scale, from complete disagreement to complete agreement. Detailed in Table 4.

**Table 4:** Description of AI access

Item	Median [IQR]	Mode	Scale
Journals	0.00 [0.00, 1.00]	0.00	AI source
Online Courses	0.00 [0.00, 1.00]	0.00	
Hospital Training	0.00 [0.00, 0.00]	0.00	
Social Media	1.00 [1.00, 1.00]	1.00	
Conferences	0.00 [0.00, 0.00]	0.00	
Colleague Discussions	1.00 [0.00, 1.00]	1.00	
News & Websites	1.00 [0.00, 1.00]	1.00	
Guidelines	0.00 [0.00, 0.00]	0.00	
Books	0.00 [0.00, 0.00]	0.00	
Vendor Docs	0.00 [0.00, 0.00]	0.00	
Other	0.00 [0.00, 0.00]	0.00	
Reading Articles	2.00 [2.00, 3.00]	2.00	AI Exposure & Learning
Access & Use	3.00 [2.00, 4.00]	4.00	
Active Seeking	3.00 [3.00, 4.00]	4.00	
AI Exposure & Learning	3.00 [2.33, 3.92]	2.67	
Understanding	3.00 [2.00, 3.00]	3.00	AI Trust & Expectations
Effective Use	3.00 [2.00, 4.00]	3.00	
Future Importance	4.00 [3.00, 5.00]	5.00	
AI Trust & Expectations	3.33 [2.67, 4.00]	3.33	

### Psychiatrists’ self-efficacy and trust in AI

This subsection evaluates psychiatrists’ self-reported confidence in understanding and using AI in clinical practice. Responses were rated on a 1–5 scale, from strongly disagree to strongly agree. For the statement *“I have a good understanding of currently available AI technologies”*, most psychiatrists indicated neutrality (mean = 2.9, mode = 3). Similarly, for the statement *“I believe I can effectively use AI tools to assist in my work”*, neutrality was again the most common response (mean = 3.17, mode = 3).

This subsection explores psychiatrists’ trust in the future role of AI in mental healthcare. Most respondents agreed that AI will become an important component of mental health care in the coming years (mean = 3.92, mode = 5). Psychiatrists who had received AI training also showed significantly higher trust (Mann-Whitney U = 587.000, *p* = 0.024), with a median of 5 (IQR: 4–5), compared to 4 (IQR 3–5) for those without training.

### Psychiatrists’ design expectations for AI

**Fig. 2:**
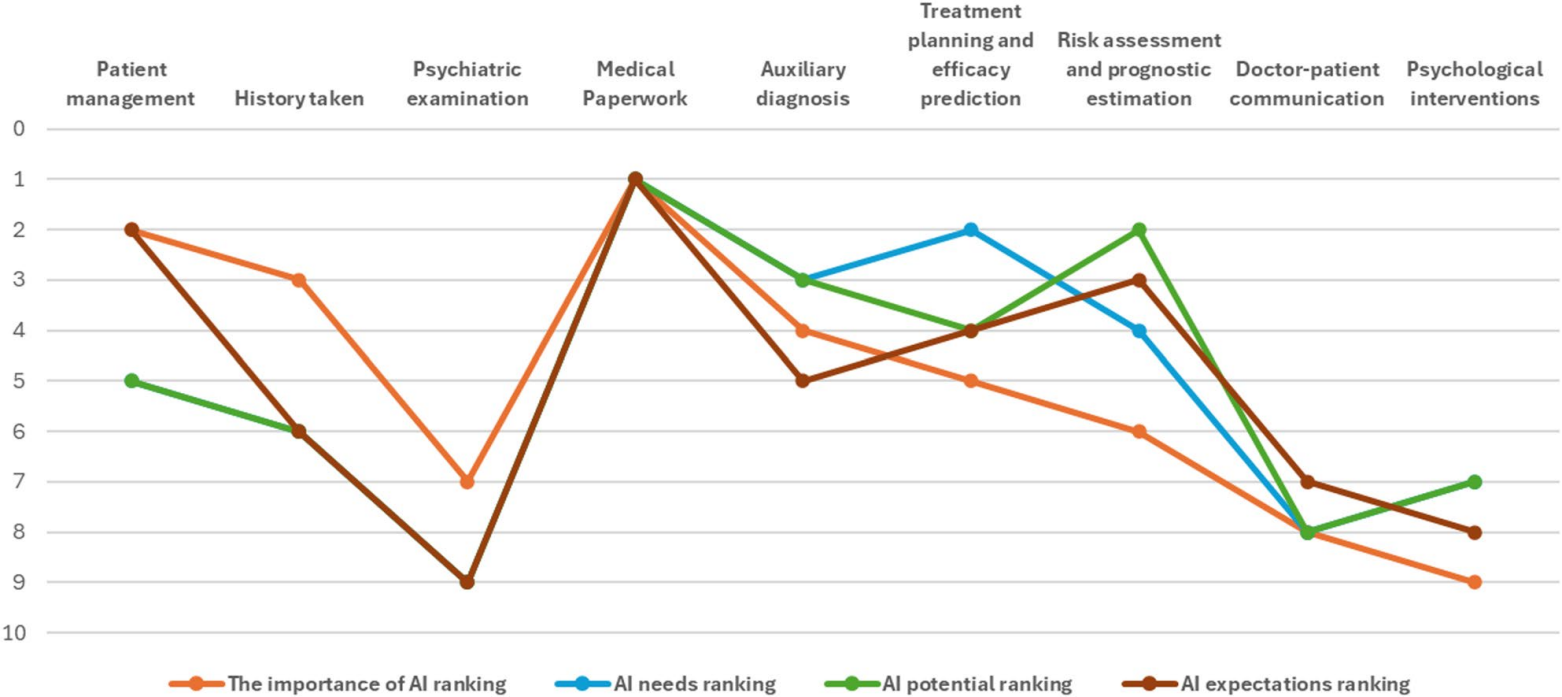
Comparative rankings of AI importance, needs, potential, and expectations across nine psychiatric medical scenarios (lower scores indicate higher priority)

To better understand psychiatrists’ priorities and attitudes toward AI applications, the study assessed their views on the importance, demand, potential, and expectations of AI across a range of clinical scenarios. Figure 2 illustrates psychiatrists’ rankings of AI applications across clinical scenarios, showing strong alignment in perceived importance, demand, potential, and willingness to adopt, with medical documentation and treatment planning consistently rated highest.

Table 5 represents the overview demand, potential, and expectations across scenarios for AI. Psychiatrists rated the relevance of AI across three professional domains: clinical care, science communication, and management. Clinical practice was prioritized most highly (mean rank = 1.34), followed by science communication (2.05), and management (2.60).

Regarding specific psychiatric medical scenarios, psychiatrists rank the importance of AI applications from high to low as follows: medical documentation writing (3.29), patient management (3.3), medical history collection (3.6). Psychiatrists also rated the need for AI in these scenarios using a 5-point scale (1 = no need, 5 = very high need). The highest-rated needs were medical documentation drafting, treatment planning and efficacy prediction, assisted diagnosis. Perceived potential of AI across scenarios, rated from 1 (very limited) to 5 (very significant), followed a similar trend. Medical documentation was rated highest, followed by risk assessment and prognosis estimation, diagnostic assistance.

**Table 5:** Description of AI demand, potential, and expectations across scenarios

	Demand		Potential		Optimization	
Item	Median[IQR]	Mode	Median[IQR]	Mode	Median[IQR]	Mode
Patient Management	4.00 [3.00, 5.00]	4.00	4.00 [3.00, 5.00]	5.00	4.00 [4.00, 5.00]	5.00
Medical History Collection	3.00 [3.00, 4.00]	3.00	4.00 [3.00, 5.00]	5.00	4.00 [3.00, 5.00]	5.00
Mental Status Examination	3.00 [2.00, 4.00]	3.00	3.00 [3.00, 5.00]	3.00	4.00 [3.00, 5.00]	5.00
Medical Documentation Writing	4.00 [4.00, 5.00]	5.00	5.00 [4.00, 5.00]	5.00	5.00 [4.00, 5.00]	5.00
Diagnostic Assistance	4.00 [3.00, 5.00]	4.00	4.00 [4.00, 5.00]	5.00	4.00 [3.00, 5.00]	5.00
Treatment Planning and Outcome Prediction	4.00 [3.25, 5.00]	4.00	4.00 [3.00, 5.00]	5.00	4.00 [4.00, 5.00]	5.00
Risk Assessment and Prognosis Estimation	4.00 [3.00, 5.00]	4.00	4.00 [4.00, 5.00]	5.00	4.00 [4.00, 5.00]	5.00
Doctor-Patient Communication	3.00 [2.00, 4.00]	3.00	4.00 [2.25, 5.00]	5.00	4.00 [3.00, 5.00]	5.00
Psychological Interventions	3.00 [2.00, 4.00]	4.00	4.00 [3.00, 5.00]	4.00	4.00 [3.00, 5.00]	5.00
Total	3.72 [3.00, 4.22]	3.78	3.89 [3.25, 4.64]	5.00	4.00 [3.36, 4.89]	5.00
F1	4.00 [3.33, 4.67]	5.00	4.00 [3.50, 4.83]	5.00	4.17 [3.67, 5.00]	5.00
F2	3.75 [3.00, 4.25]	4.00	3.67 [2.67, 4.33]	5.00	3.67 [2.67, 5.00]	5.00
F3	3.50 [2.50, 4.00]	4.00	-	-	-	-

#### Influencing factors on AI importance, demand, potential, and expectations

We analyses these inflencing factors: sex, age, educational background, doctor titles, whether recieving AI training, whether is hospital levels. Psychiatrists were divided into 5 groups by age ranges (18–23, 24–30, 31–40, 41–50, over 50 years old).Psychiatrists were divided into four groups according to their educational background: associate degree, bachelor’s degree, master’s degree, and doctoral degree. Psychiatrists were grouped into four categories based on their titles: resident physicians, attending physicians, associate chief physicians, and chief physicians.

**Fig. 3:**
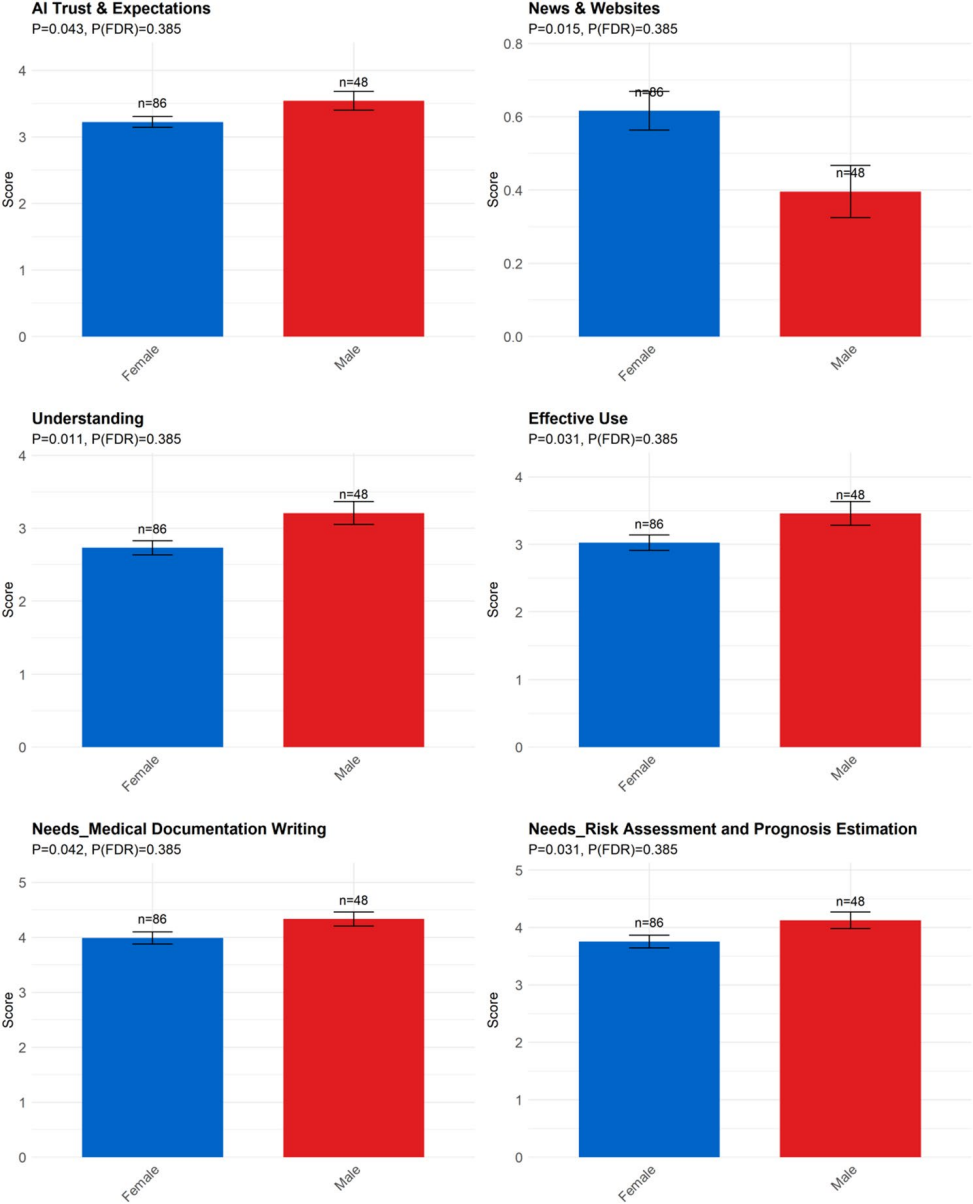
Differences among different sex group. Note: AI Trust & Expectations: AI Trust & Expectations; News & Websites: News media and websites; Understanding: I have a good understanding of the AI technologies currently available; Effective Use: I believe I can effectively use AI tools to support my work; Needs Medical Documentation Writing: Medical Documentation Writing; Needs Risk Assessment and Prognosis Estimation: Risk Assessment and Prognosis Estimation

Group difference analyses were conducted to examine how AI perception varied across different demographic characteristics. Significant findings are summarized below:

Gender (Fig. 3): Significant differences were observed between male and female participants in AI Trust & Expectations and the utilization of News & Websites as information sources. Gender also influenced self-reported Understanding of AI technologies and the belief in their Effective Use. Regarding specific clinical needs, significant variations were found in Medical Documentation Writing and Risk Assessment and Prognosis Estimation.

**Fig. 4:**
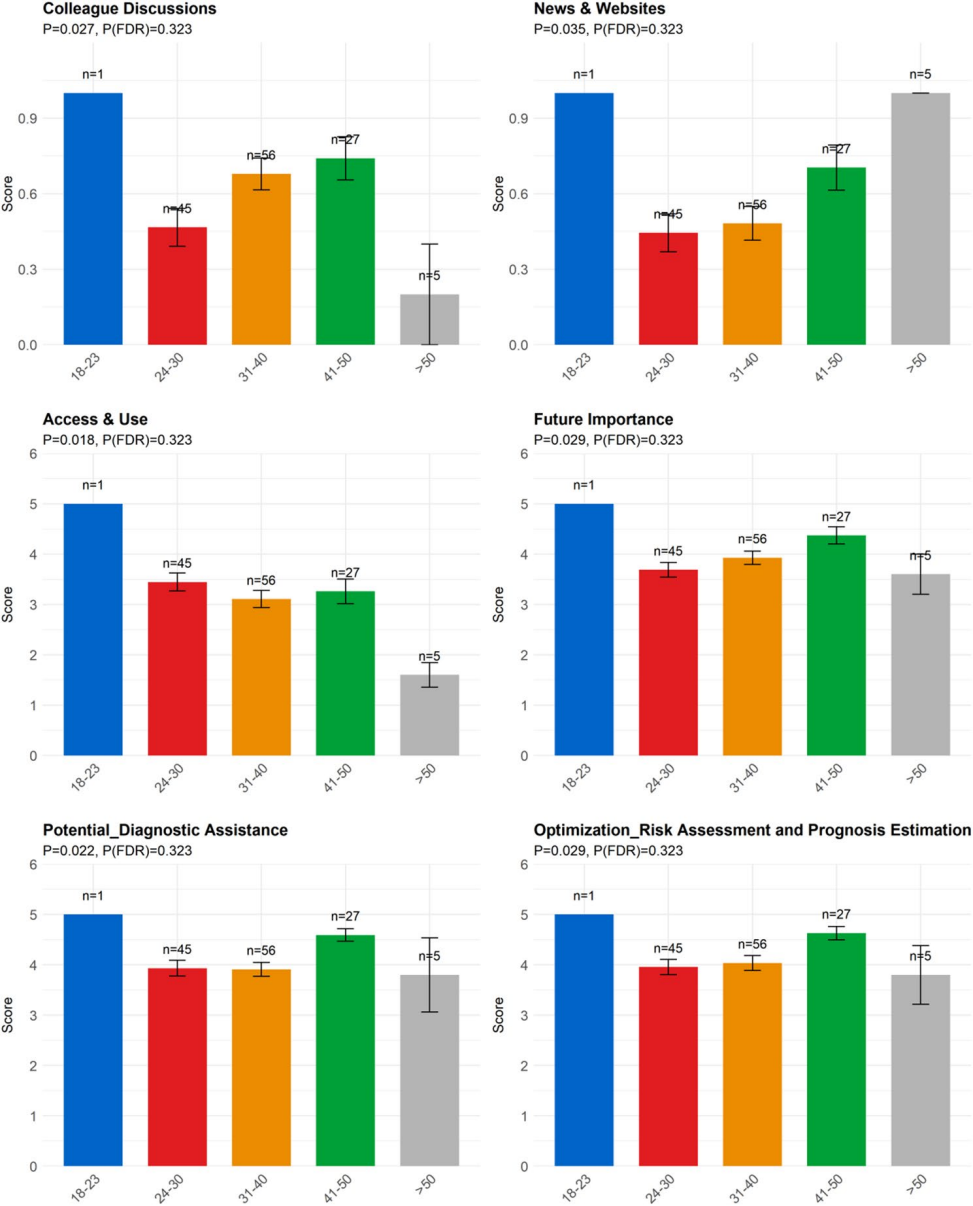
Differences among different age group. Note: Colleague Discussions: Communication and discussions with colleagues; News & Websites: News media and websites; Access & Use: In my daily work, I can access and use AI technologies; Future Importance: I believe that AI technologies will become an important part of mental health care in the coming years; Potential Diagnostic Assistance: Diagnostic Assistance; Optimization Risk Assessment and Prognosis Estimation: Risk Assessment and Prognosis Estimation

Age (Fig. 4): Age groups differed significantly in their engagement with Colleague Discussions and News & Websites, as well as their reported Access & Use of AI technologies in daily work. Beliefs regarding the Future Importance of AI in mental health care also varied by age. In terms of specific applications, significant differences were noted in the perceived potential for Diagnostic Assistance and optimization needs for Risk Assessment and Prognosis Estimation.

**Fig. 5:**
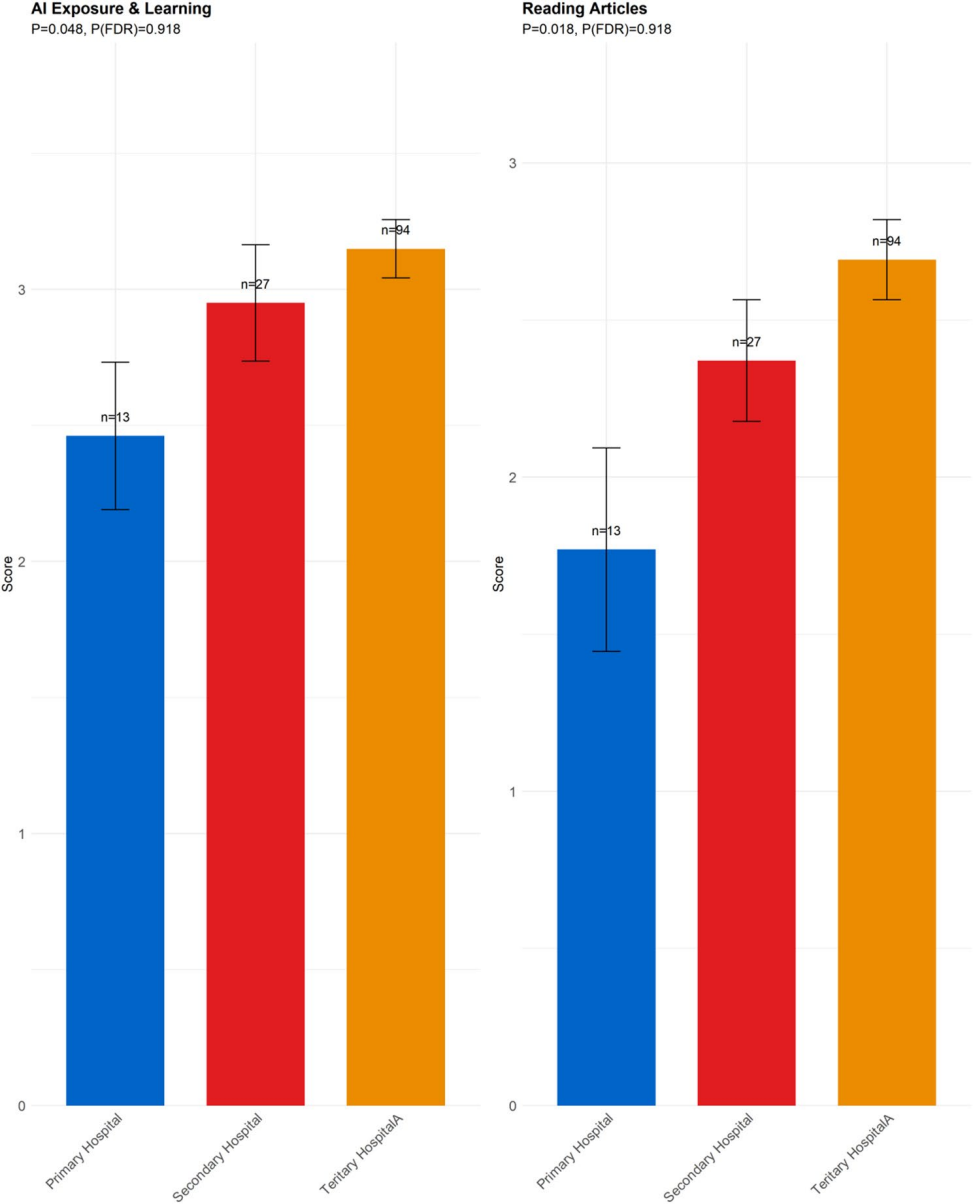
Differences among different hospital level group. Note: AI Exposure & Learning: AI Exposure & Learning; Reading Articles: In the past year, I have frequently read articles or reports on the application of AI in the medical field

Hospital Level (Fig. 5): Participants from different hospital levels showed significant variation in overall AI Exposure & Learning and the frequency of Reading Articles on medical AI applications.

**Fig. 6:**
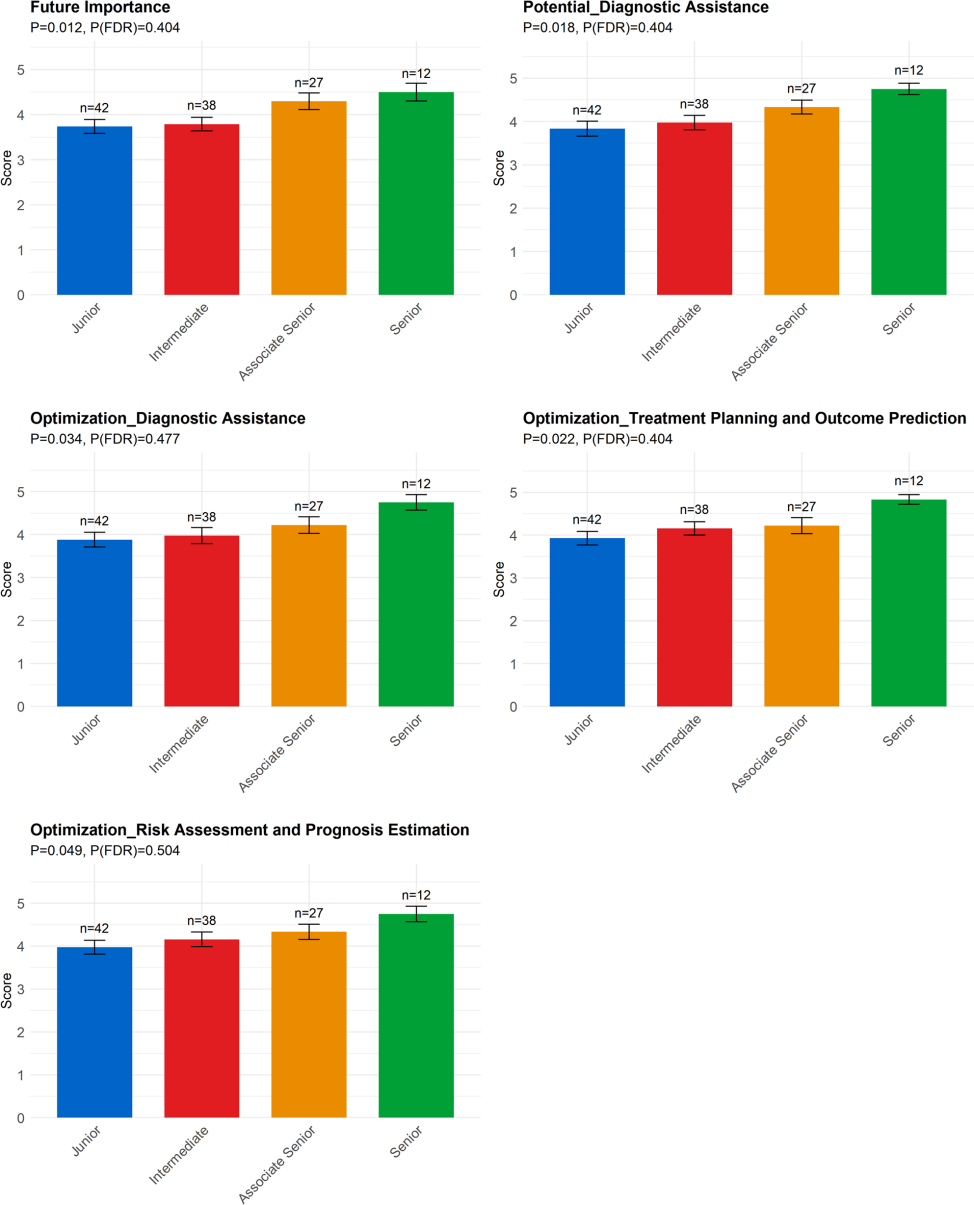
Differences in different titles group. Note: Future Importance: I believe that AI technologies will become an important part of mental health care in the coming years; Potential_Diagnostic Assistance: Diagnostic Assistance; Optimization_Diagnostic Assistance: Diagnostic Assistance; Optimization_Treatment Planning and Outcome Prediction: Treatment Planning and Outcome Prediction; Optimization_Risk Assessment and Prognosis Estimation: Risk Assessment and Prognosis Estimation

Professional Title (Fig. 6): Professional titles significantly influenced views on the Future Importance of AI. Differences were also observed in the perceived potential for Diagnostic Assistance, as well as optimization expectations for Diagnostic Assistance, Treatment Planning and Outcome Prediction, and Risk Assessment and Prognosis Estimation.

**Fig. 7:**
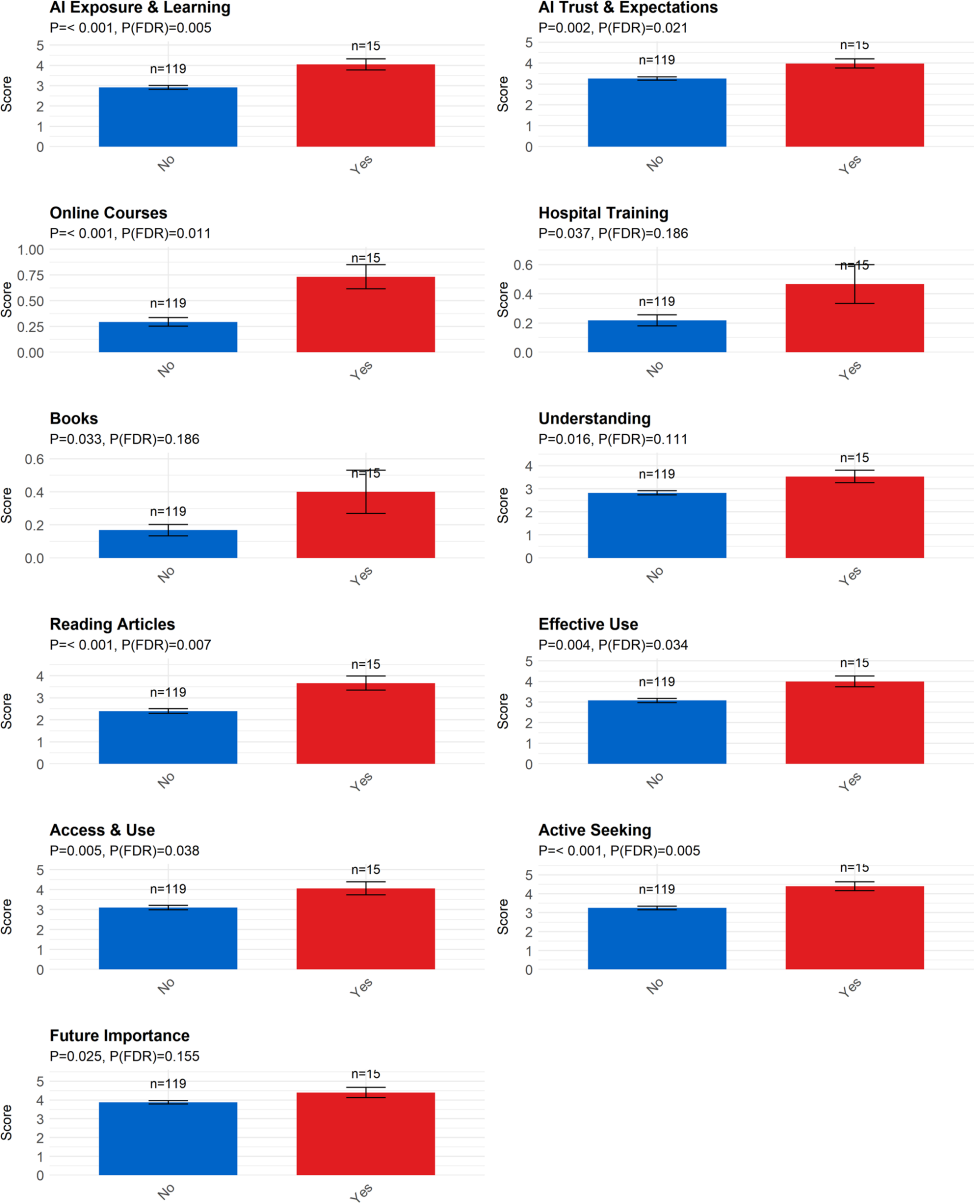
Differences among different AI Training Experience group. Note: AI Exposure & Learning: AI Exposure & Learning; AI Trust & Expectations: AI Trust & Expectations; Online Courses: Online courses and training programs; Hospital Training: In-house training at hospitals; Books: Books (including e-books); Understanding: I have a good understanding of the AI technologies currently available; Reading Articles: In the past year, I have frequently read articles or reports on the application of AI in the medical field; Effective Use: I believe I can effectively use AI tools to support my work; Access & Use: In my daily work, I can access and use AI technologies; Active Seeking: I actively seek out information about AI technologies to improve my professional competence; Future Importance: I believe that AI technologies will become an important part of mental health care in the coming years

AI Training Experience (Fig. 7): Participation in AI training had a widespread impact on AI perception. Significant differences were found in AI Exposure & Learning, AI Trust & Expectations, and the use of learning channels such as Online Courses, Hospital Training, and Books. Training experience also significantly affected self-assessed Understanding, Reading Articles, Effective Use, Access & Use, Active Seeking behaviors, and beliefs about Future Importance.

**Fig. 8:**
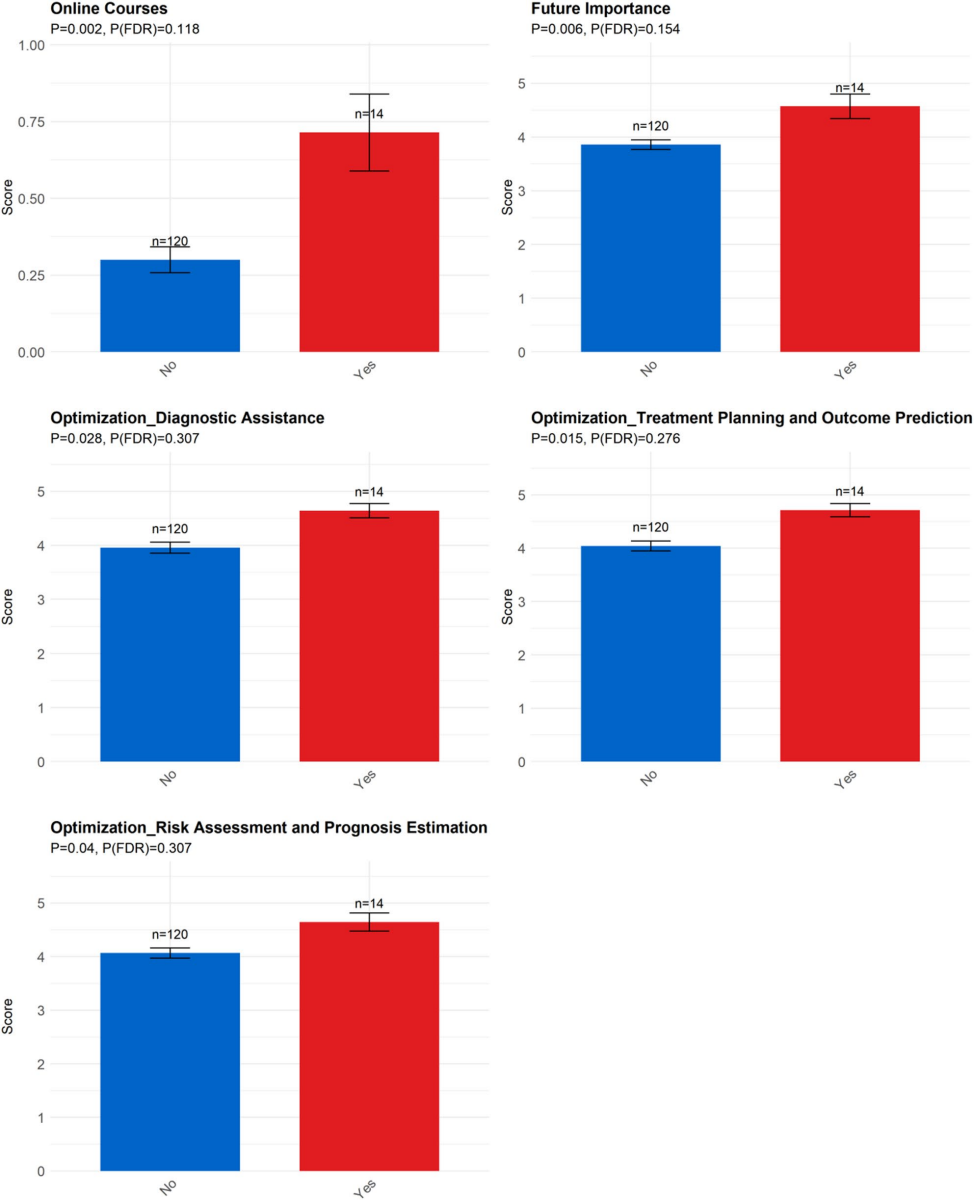
Differences among different Department Director Status group. Note: Online Courses: Online courses and training programs; Future Importance: I believe that AI technologies will become an important part of mental health care in the coming years; Optimization_Diagnostic Assistance: Diagnostic Assistance; Optimization_Treatment Planning and Outcome Prediction: Treatment Planning and Outcome Prediction; Optimization_Risk Assessment and Prognosis Estimation: Risk Assessment and Prognosis Estimation

Department Director Status (Fig. 8): Being a department director was associated with significant differences in the use of Online Courses and beliefs in Future Importance. Directors also differed in their optimization needs for Diagnostic Assistance, Treatment Planning and Outcome Prediction, and Risk Assessment and Prognosis Estimation.

## Discussion

This section discusses psychiatrists’ readiness for AI, as well as how factors such as background, experience, and professional roles influence readiness through their impact on access, self-efficacy, trust, and design expectations. Overall, psychiatrists reported moderate self-efficacy in understanding and using AI, high trust in AI’s future role in mental healthcare, and clear design priorities. They consistently valued AI most in tasks such as medical documentation, treatment planning, diagnostic assistance, and risk assessment, indicating strong interest in tools that enhance efficiency and support complex decision-making. In contrast, applications involving psychological interventions and doctor–patient communication were rated lower, suggesting that psychiatrists see these interpersonal domains as less amenable to AI. Taken together, the findings show that psychiatrists are receptive to AI adoption, particularly where it can streamline workflows and augment clinical judgement, while maintaining expectations that human-centered care remains essential. This study demonstrates that psychiatrists have gained varying levels of understanding and knowledge about AI through different means, which in turn has influenced their belief in AI capabilities and their confidence in using AI themselves. This also affects their comprehension of AI application in various medical scenarios. Additionally, some personal characteristics of doctors, such as gender, age, and position, significantly influence their perspectives on AI.

### Psychiatrists’ AI access

As reported in Section ***Overview of AI access***, psychiatrists primarily acquire AI-related knowledge from fragmented sources such as social media, peer communication, and news media. In contrast, fewer engage with systematic learning resources like academic journals, online courses, or formal training programs. Books, industry conferences, technical documentation, and government reports are even less commonly used. This phenomenon is also commonly observed in investigative studies conducted in other regions [41, 42]. Interestingly, most psychiatrists show a high level of initiative in learning about AI and have opportunities to encounter and use AI technologies in their daily work. Our study presents greater opportunities for AI access compared to the survey conducted at Marburg University Hospital in Germany [43]. However, the proportion of those who read related articles and reports is relatively low. This may be because acquiring knowledge through fragmented sources is more convenient and accessible than undergoing systematic training and learning through media and communication channels is more appealing than self-studying specialized knowledge. For AI professionals, it is hoped that future AI applications can be designed to be more user-friendly, lower the entry barriers, enhance promotion efforts, and improve professionalism, particularly in areas such as social media.

As reported in Section ***Group Differences in AI Access***, department heads more frequently obtained AI knowledge through news media and training courses, while non-managerial doctors relied more on academic literature. Our findings suggest that under the macro decision-making of managers, doctors in non-managerial positions need to pay more attention to the details of these knowledge and technologies, which often come from professional journals and academic papers. This reflects that the detailed parts of current AI knowledge are more easily obtained by ordinary doctors, while the more macro and influential knowledge is more easily obtained by directors. This reflects that there is still a certain lack of basic popularization of AI knowledge, and accurate content has not been objectively conveyed to the hands of department decision-makers. This suggests that news media should balance professionalism and objectivity when promoting AI technologies.

Although some institutions provide training opportunities, only around 10% of psychiatrists participated in AI-related workshops. Notably, department heads were much more likely to receive such training: two-thirds had engaged in formal learning, compared to one-third of non-managers (see Section ***Group Differences in AI Access***). This suggests a need to strengthen AI training programs for frontline psychiatrists and managers.

Hospital tier also appears to influence AI engagement. Psychiatrists from tertiary hospitals were more likely to spend time reading medical AI literature than those in primary care settings. This reflects different professional responsibilities: community-based psychiatrists often focus on routine care, while those in tertiary hospitals manage complex cases and pursue academic development. This also reflects that current AI technology is more inclined towards assisting with the challenging topics in psychiatry, while further development is still required for its functionality in grassroots medical services. The broader application of AI is closely tied to psychiatrists’ self-efficacy and trust in using AI.

### Psychiatrists’ self-efficacy and trust in AI

Psychiatrists’ confidence in using AI varies notably by gender, age, and training experience (see Section ***Self-Efficacy***). This contrasts with a survey from Bahrain [44], which found that doctors’ motivation was unrelated to factors such as gender, age and knowledge of AI. Our survey indicates that male and younger psychiatrists, as well as those who had completed AI-related training, reported higher self-efficacy. In contrast, female psychiatrists and those over 50 were more likely to report uncertainty or lower confidence in applying AI tools. Our findings regarding gender differences are consistent with those reported by Naiseh et al. in their sample from the United Kingdom [45] and Doraiswamy et al.‘s study from 22 countries worldwide [46], such as males exhibiting higher positive attitudes and lower negative attitudes compared to females. This may suggest that there is some inequity in the accessibility and usability of AI, potentially influenced by differences in cognitive learning styles and stereotypical self-perceptions, which result in a lack of confidence among some women and older individuals in mastering AI technologies. Self-efficacy plays a crucial role in shaping attitudes towards AI, with higher levels of self-efficacy associated with more positive attitudes and enhanced well-being [45]. On one hand, it is hoped that AI will provide more tailored services for women and older adults in future design; on the other hand, it is anticipated that relevant training programs can assist these groups in building greater confidence.

Trust in AI was generally positive, which is consistent with previous research findings [44, 46]. Most psychiatrists agreed that AI will become an important part of mental healthcare in the near future. From a psychological perspective, psychiatrists trust in AI is initially a form of instrumental rationality, wherein AI is perceived as an enhancer capable of efficiently managing administrative tasks and supporting information integration—aligning with physicians needs to improve work efficiency and decision-making quality. Simultaneously, as a controllable auxiliary agent, AI compensates for knowledge gaps, thereby reinforcing clinicians’ sense of professional mastery. At the organizational level, the pressing demands for improved efficiency and optimized resource allocation within healthcare systems render AI a viable solution to address imbalances between service supply and demand. Moreover, the principles of standardization and evidence-based practice position AI as a tool to promote diagnostic and therapeutic homogenization. Additionally, peer influence within the professional community further strengthens physicians’ trust in AI. Department heads expressed especially strong confidence in their future role, possibly reflecting their greater access to strategic information and exposure to optimistic media narratives. However, as noted in Section ***Overview of AI access***, the heavy reliance on media may lead to overestimated expectations. This underscores the need for more balanced, evidence-based communication about AI’s capabilities and limitations, particularly for clinical leaders guiding institutional decisions.

Furthermore, the importance of training becomes evident. The paragraph on Self-Efficacy and Trust in AI indicates that psychiatrists who have received AI-related training exhibit higher levels of self-efficacy and trust. This may be attributed to the findings reported by Naiseh et al., it was mentioned that AI competency serves as a mediator, with increased competence fostering greater confidence and positivity towards AI [45].

### Psychiatrists’ design expectations for AI

Psychiatrists reported the highest demand for AI in clinical treatment, followed by science communication and administrative management (see Section ***Overview of Importance***,*** Demand***,*** Potential***,*** and Expectations across Scenarios***). In terms of the importance, usage requirements, potential, and expectations across various medical scenarios, medical documentation consistently ranked first in importance, demand, potential, and willingness to adopt. Consistent with the findings of Blease [47] and Doraiswamy [46], administrative tasks are identified as a primary advantage of AI tools, such as “documentation becoming more efficient”. This differs from the findings of Fischer et al., who reported that clinicians prefer AI-assisted prediction of symptom trajectories and patient monitoring [43]. This likely reflects the burden of documentation-related tasks in psychiatric practice in China, which contribute significantly to burnout [48]. Currently, ChatGPT has been applied to some extent in psychiatric practice, primarily assisting psychiatrists in completing routine tasks to alleviate the burden of clinical documentation, communication, and research [49]. This is also the primary need highlighted in this study, such as transcribing and summarizing medical dialogues, generating standard or customized medical records, which can then be reviewed and modified by physicians for use as official medical documentation, thereby reducing clinical pressure.

Auxiliary diagnosis also received high interest, especially from department heads and psychiatrists in tertiary hospitals, who typically manage complex or ambiguous cases. However, the reliability of AI in psychiatric diagnosis remains limited. In recent years, China has conducted numerous studies developing diagnostic functions for mental disorders using AI technologies, such as Crossmodally Audiovisual Emotional Integration for diagnosing depression [50], the use of fMRI to differentiate between healthy individuals and patients with psychiatric disorders [13], and AI-powered integration of multimodal imaging in precision medicine for neuropsychiatric conditions [51]. However, current AI technologies still require improvements in interpretability and further validation. Unlike many other specialties, psychiatry lacks objective diagnostic markers and relies heavily on narrative information and clinician judgment. The existing AI systems still lacks validated accuracy in these contexts, and effective performance evaluation methods are lacking. This makes diagnostic support a promising but currently underdeveloped application area.

By contrast, psychiatrists showed relatively low expectations for AI in psychiatric assessment, doctor-patient communication, and psychotherapy. These domains involve nuanced interpersonal skills, empathy, and emotional presence, which current AI systems are ill-equipped to replicate. AI tools predominantly rely on standardized templates and preset al.gorithms, enabling them to provide appropriate responses during conversations but still appearing rigid in demonstrating empathy and interpreting implicit meanings [52]. They also face challenges in maintaining appropriate silences or guiding deeper dialogues. Additionally, non-verbal expressions from patients with mental disorders carry significant value; eye contact, facial expressions, tone of voice, posture, and gestures can all convey their inner experiences. Current AI technologies encounter difficulties in receiving, processing, and expressing such signals. Furthermore, concerns about data privacy, accountability, and patient trust also limit enthusiasm for AI use in direct patient interaction [53]. Some psychiatrists expressed fears of professional devaluation or replacement [54], especially in tasks closely tied to therapeutic identity—which may also contribute to skepticism.

In summary, psychiatrists are cautiously optimistic about AI and demonstrate a relatively high level of readiness. In terms of access, most have opportunities to encounter and utilize AI technologies in their daily practice and are accustomed to acquiring AI-related knowledge through social media, peer communication, and news media. However, structured learning and formal training opportunities remain limited. Regarding self-efficacy, most psychiatrists rate their competence at a moderate level, with higher self-assessments observed among male and younger physicians. With respect to trust, psychiatrists generally hold positive attitudes toward AI, with department heads expressing greater optimism. In terms of Design Expectations, psychiatrists are more enthusiastic about using AI to reduce the administrative burden. However, human-centered roles remain sensitive territory. Ethical safeguards, user-driven design, and ongoing clinical involvement in development will be essential to ensuring that future AI tools genuinely support rather than displace psychiatric professionals. The development of AI technology in psychiatry relies not only on expanding education and training for physicians but also on actively involving psychiatrists in the development, implementation, and operation of AI software while ensuring continuous monitoring. Future efforts should focus on promoting broader accessibility, improving the quality and credibility of information dissemination, and ensuring that AI technologies align with clinical realities. Integrating multimodal data and embedding continuous user feedback will be essential for building AI systems that genuinely support psychiatric practice and improve patient care.

## Practical implications

Based on these findings, several practical strategies may enhance psychiatrists’ readiness for AI in clinical practice. First, structured and tier-specific AI training programs, particularly for clinician groups in our sample who tended to report lower digital confidence, including those with extensive practice experience, could strengthen competence and self-efficacy. Second, balanced and evidence-based communication from hospitals and professional associations is essential to promote realistic expectations and reinforce trust, especially given clinicians’ frequent reliance on media sources for AI information. Third, AI developers should adopt user-centered design principles that align with psychiatrists’ priorities, such as reducing documentation burden, supporting complex diagnostic reasoning, and integrating seamlessly into existing workflows. Finally, involving psychiatrists throughout development, evaluation, and implementation can help ensure that AI tools remain clinically relevant, ethically sound, and supportive of professional autonomy.

## Strengths and limitations

This study recruited psychiatrists from hospitals of different levels, regions, and professional titles and positions, making the sample relatively representative and facilitating a comprehensive understanding of AI-related needs among psychiatrists in China. Furthermore, this study discussed multiple perspectives in AI-driven psychiatry applications, including access, self-efficacy, trust, and design expectations, identifying various practical needs in clinical practice and providing directions for future optimization of artificial intelligence technologies.

Nonetheless, this study still has several limitations. The findings reflect only the perspectives of psychiatrists, which may differ from those of physicians in other clinical specialties. Additionally, although we identified the needs and expectations of psychiatrists regarding AI, we were unable to propose specific solutions, which remains further development by professionals in related fields in the future.

## Conclusion

Psychiatrists are cautiously optimistic about AI and demonstrate a relatively high level of readiness, especially when it reduces the administrative burden. Further foundational promotion of AI requires simplification in application design to enhance accessibility and trust among diverse user groups. Social media communication may benefit from greater emphasis on professionalism and objectivity, and expanding training opportunities could further support psychiatrists’ engagement with AI. The involvement of psychiatrists in the development, implementation, and operational processes of AI technologies is beneficial for delivering high-quality clinical services.

## Supplementary Information

Below is the link to the electronic supplementary material.

Supplementary Material 1

Supplementary Material 2

## Data Availability

The datasets used and/or analyzed during the current study are available from the corresponding author on reasonable request.

## Abbreviations

AI: Artificial intelligence
LLM: Large language models
