# Supplementary material for: Understanding psychiatrist readiness for AI: a study of access, self-efficacy, trust, and design expectations
Source: BMC Health Serv Res. 2026 Jan 17;26:218. doi: 10.1186/s12913-026-14010-6 (PMC12895999; doi:10.1186/s12913-026-14010-6)
Supplement: Supplementary file 1 — Supplementary Material 1 [file 12913_2026_14010_MOESM1_ESM.docx]

Psychiatric AI Questionnaire-Clinical Needs Assessment

We are conducting a study *Investigation of AI Requirements and Application Optimization in Psychiatry.* We invite you to complete this questionnaire. Please confirm that you have read and understood the information provided and that you voluntarily agree to participate.

1. Please select your city.
2. What is the level of your hospital?
3. Primary Hospital
4. Secondary Hospital
5. Tertiary Hospital
6. Gender
7. Male
8. Female
9. prefer not to disclose
10. Age
11. 18-23 years old
12. 24-30 years old
13. 31-40 years old
14. 41-50 years old
15. over 50 years old
16. prefer not to disclose
17. Education Background
18. Associate Degree
19. Bachelor's Degree
20. Master's Degree
21. Doctoral Degree
22. Prefer Not to Disclose
23. Position
24. Psychiatrist
25. Researcher
26. Teaching Staff
27. (Deputy) Ward Director
28. Engaged in the current profession for ___ years.
29. My knowledge of AI technology comes from
30. Academic journals and research papers
31. Online courses and training programs
32. In-house training at hospitals
33. Social media platforms
34. Industry conferences and exhibitions
35. Communication and discussions with colleagues
36. News media and websites
37. Guidelines and reports issued by government agencies and industry associations
38. Books (including e-books)
39. Product specifications and technical documentation provided by vendors
40. Other______________________
41. Please rate the following statements on a scale from 1 to 5 (1 = strongly disagree, 5 = strongly agree)
42. I have a good understanding of the AI technologies currently available.
43. In the past year, I have frequently read articles or reports on the application of AI in the medical field.
44. I believe I can effectively use AI tools to support my work.
45. In my daily work, I can access and use AI technologies.
46. I actively seek out information about AI technologies to improve my professional competence.
47. I believe that AI technologies will become an important part of mental health care in the coming years.
48. Have you participated in any training sessions or workshops related to AI technology?
49. Yes, please describe the activity content ____________.
50. No.
51. How helpful were these AI technology training activities in your understanding of AI technology? (1 = No help, 5 = Very helpful)
52. Please rank the following scenarios according to their importance in your work:
53. Medical Care
54. Science Popularization
55. Departmental Management
56. Please rank the importance of applying AI technology in the following psychiatric clinical scenarios:
57. Patient Management
58. Medical History Collection
59. Mental Status Examination
60. Medical Documentation Writing
61. Diagnostic Assistance
62. Treatment Planning and Outcome Prediction
63. Risk Assessment and Prognosis Estimation
64. Doctor-Patient Communication
65. Psychological Interventions
66. Others ____________.
67. Please rate the demand for AI in each of the above medical scenarios on a scale of 1 to 5. (1 = No demand at all, 5 = Very high demand)
68. Please rate the potential of AI in the medical scenarios on a scale of 1 to 5. (1 = very limited, 5 = very significant)
69. Are you willing to try new AI tools to improve the above medical scenarios? (1=Completely unwilling, 5=Very willing)
