# Supplementary material for: Understanding psychiatrist readiness for AI: a study of access, self-efficacy, trust, and design expectations
Source: BMC Health Serv Res. 2026 Jan 17;26:218. doi: 10.1186/s12913-026-14010-6 (PMC12895999; doi:10.1186/s12913-026-14010-6)
Supplement: Supplementary file 2 — Supplementary Material 2 [file 12913_2026_14010_MOESM2_ESM.docx]

Table S1：Group differences between sex

| **Item** | **Group** | **Descriptive Statistics** | **Statistic** | **Raw P-value** | **P-value (FDR)** | **Post-hoc Test** |
| --- | --- | --- | --- | --- | --- | --- |
| AI Exposure & Learning | Female (n=86) | 2.67 (2.33-3.67) | Z=1691 | 0.083 | 0.385 |  |
|  | Male (n=48) | 3.26±1.1 |  |  |  |  |
| AI Trust & Expectations | Female (n=86) | 3.33 (2.67-3.67) | Z=1630 | 0.043 | 0.385 |  |
|  | Male (n=48) | 3.54±0.97 |  |  |  |  |
| Needs_Clinical_Decision | Female (n=86) | 4 (3-4.67) | Z=1767.5 | 0.164 | 0.579 |  |
|  | Male (n=48) | 4 (3.67-5) |  |  |  |  |
| Needs_Routine_Tasks | Female (n=86) | 3.75 (3-4.25) | Z=1933 | 0.543 | 0.779 |  |
|  | Male (n=48) | 3.75 (3.19-4.25) |  |  |  |  |
| Needs_Interaction | Female (n=86) | 3.5 (2.5-4) | Z=2224 | 0.455 | 0.758 |  |
|  | Male (n=48) | 3.25 (2-4) |  |  |  |  |
| Medical Needs | Female (n=86) | 3.78 (3-4.22) | Z=2017.5 | 0.831 | 0.929 |  |
|  | Male (n=48) | 3.67±0.77 |  |  |  |  |
| Potential_Clinical_Efficiency | Female (n=86) | 4 (3.5-4.83) | Z=1929 | 0.529 | 0.779 |  |
|  | Male (n=48) | 4.17 (3.5-5) |  |  |  |  |
| Potential_Humanistic_Care | Female (n=86) | 3.67 (2.67-4.33) | Z=2184.5 | 0.575 | 0.779 |  |
|  | Male (n=48) | 3.38±1.14 |  |  |  |  |
| Medical Potential | Female (n=86) | 3.89 (3.22-4.67) | Z=2037.5 | 0.904 | 0.935 |  |
|  | Male (n=48) | 3.78 (3.5-4.47) |  |  |  |  |
| Optimization_Clinical_Efficiency | Female (n=86) | 4.17 (3.54-5) | Z=2125.5 | 0.773 | 0.921 |  |
|  | Male (n=48) | 4.17 (3.67-5) |  |  |  |  |
| Optimization_Humanistic_Care | Female (n=86) | 4 (3-5) | Z=2454.5 | 0.067 | 0.385 |  |
|  | Male (n=48) | 3.33 (2.33-4.17) |  |  |  |  |
| Medical Optimization | Female (n=86) | 4 (3.33-5) | Z=2303 | 0.265 | 0.593 |  |
|  | Male (n=48) | 3.86±0.82 |  |  |  |  |
| Journals | Female (n=86) | 0 (0-1) | Z=1814 | 0.169 | 0.579 |  |
|  | Male (n=48) | 0 (0-1) |  |  |  |  |
| Online Courses | Female (n=86) | 0 (0-1) | Z=2029 | 0.846 | 0.929 |  |
|  | Male (n=48) | 0 (0-1) |  |  |  |  |
| Hospital Training | Female (n=86) | 0 (0-1) | Z=2186 | 0.45 | 0.758 |  |
|  | Male (n=48) | 0 (0-0) |  |  |  |  |
| Social Media | Female (n=86) | 1 (1-1) | Z=2201 | 0.348 | 0.722 |  |
|  | Male (n=48) | 1 (1-1) |  |  |  |  |
| Conferences | Female (n=86) | 0 (0-0) | Z=2142 | 0.56 | 0.779 |  |
|  | Male (n=48) | 0 (0-0) |  |  |  |  |
| Colleague Discussions | Female (n=86) | 1 (0-1) | Z=2333 | 0.141 | 0.579 |  |
|  | Male (n=48) | 1 (0-1) |  |  |  |  |
| News & Websites | Female (n=86) | 1 (0-1) | Z=2519 | 0.015 | 0.385 |  |
|  | Male (n=48) | 0 (0-1) |  |  |  |  |
| Guidelines | Female (n=86) | 0 (0-0) | Z=2132 | 0.554 | 0.779 |  |
|  | Male (n=48) | 0 (0-0) |  |  |  |  |
| Books | Female (n=86) | 0 (0-0) | Z=2085 | 0.89 | 0.935 |  |
|  | Male (n=48) | 0 (0-0) |  |  |  |  |
| Vendor Docs | Female (n=86) | 0 (0-0) | Z=2161 | 0.448 | 0.758 |  |
|  | Male (n=48) | 0 (0-0) |  |  |  |  |
| Other | Female (n=86) | 0 (0-0) | Z=2069 | 0.935 | 0.935 |  |
|  | Male (n=48) | 0 (0-0) |  |  |  |  |
| Understanding | Female (n=86) | 3 (2-3) | Z=1547.5 | 0.011 | 0.385 |  |
|  | Male (n=48) | 3 (3-4) |  |  |  |  |
| Reading Articles | Female (n=86) | 2 (1-3) | Z=1699 | 0.082 | 0.385 |  |
|  | Male (n=48) | 3 (2-4) |  |  |  |  |
| Effective Use | Female (n=86) | 3 (2-4) | Z=1613.5 | 0.031 | 0.385 |  |
|  | Male (n=48) | 3.5 (3-4.25) |  |  |  |  |
| Access & Use | Female (n=86) | 3 (2-4) | Z=1791.5 | 0.196 | 0.579 |  |
|  | Male (n=48) | 3.5 (2.75-4) |  |  |  |  |
| Active Seeking | Female (n=86) | 3 (3-4) | Z=1697 | 0.078 | 0.385 |  |
|  | Male (n=48) | 4 (3-4) |  |  |  |  |
| Future Importance | Female (n=86) | 4 (3-5) | Z=2017.5 | 0.823 | 0.929 |  |
|  | Male (n=48) | 4 (3-5) |  |  |  |  |
| Needs_Patient Management | Female (n=86) | 4 (3-5) | Z=1820.5 | 0.238 | 0.579 |  |
|  | Male (n=48) | 4 (4-5) |  |  |  |  |
| Needs_Medical History Collection | Female (n=86) | 3 (3-4) | Z=1886.5 | 0.397 | 0.758 |  |
|  | Male (n=48) | 3.5 (3-5) |  |  |  |  |
| Needs_Mental Status Examination | Female (n=86) | 3 (2-4) | Z=2291.5 | 0.28 | 0.604 |  |
|  | Male (n=48) | 3 (2-4) |  |  |  |  |
| Needs_Medical Documentation Writing | Female (n=86) | 4 (4-5) | Z=1652.5 | 0.042 | 0.385 |  |
|  | Male (n=48) | 5 (4-5) |  |  |  |  |
| Needs_Diagnostic Assistance | Female (n=86) | 4 (3-5) | Z=1998 | 0.749 | 0.921 |  |
|  | Male (n=48) | 4 (3.75-5) |  |  |  |  |
| Needs_Treatment Planning and Outcome Prediction | Female (n=86) | 4 (3-5) | Z=1792.5 | 0.183 | 0.579 |  |
|  | Male (n=48) | 4 (4-5) |  |  |  |  |
| Needs_Risk Assessment and Prognosis Estimation | Female (n=86) | 4 (3-5) | Z=1620.5 | 0.031 | 0.385 |  |
|  | Male (n=48) | 4 (4-5) |  |  |  |  |
| Needs_Doctor-Patient Communication | Female (n=86) | 3 (2-4) | Z=2095.5 | 0.883 | 0.935 |  |
|  | Male (n=48) | 3 (2-4) |  |  |  |  |
| Needs_Psychological Interventions | Female (n=86) | 3 (3-4) | Z=2322 | 0.22 | 0.579 |  |
|  | Male (n=48) | 3 (2-4) |  |  |  |  |
| Potential_Patient Management | Female (n=86) | 4 (4-5) | Z=1938.5 | 0.54 | 0.779 |  |
|  | Male (n=48) | 4 (3-5) |  |  |  |  |
| Potential_Medical History Collection | Female (n=86) | 4 (3-5) | Z=1915 | 0.473 | 0.758 |  |
|  | Male (n=48) | 4 (3-5) |  |  |  |  |
| Potential_Mental Status Examination | Female (n=86) | 3 (3-4.75) | Z=2009.5 | 0.796 | 0.929 |  |
|  | Male (n=48) | 4 (2.75-5) |  |  |  |  |
| Potential_Medical Documentation Writing | Female (n=86) | 5 (4-5) | Z=1956.5 | 0.584 | 0.779 |  |
|  | Male (n=48) | 5 (4-5) |  |  |  |  |
| Potential_Diagnostic Assistance | Female (n=86) | 4 (4-5) | Z=2083.5 | 0.925 | 0.935 |  |
|  | Male (n=48) | 4 (3-5) |  |  |  |  |
| Potential_Treatment Planning and Outcome Prediction | Female (n=86) | 4 (3-5) | Z=1836 | 0.263 | 0.593 |  |
|  | Male (n=48) | 4.5 (3.75-5) |  |  |  |  |
| Potential_Risk Assessment and Prognosis Estimation | Female (n=86) | 4 (4-5) | Z=1815 | 0.218 | 0.579 |  |
|  | Male (n=48) | 4.5 (4-5) |  |  |  |  |
| Potential_Doctor-Patient Communication | Female (n=86) | 4 (3-5) | Z=2127 | 0.766 | 0.921 |  |
|  | Male (n=48) | 4 (2-5) |  |  |  |  |
| Potential_Psychological Interventions | Female (n=86) | 4 (3-5) | Z=2313 | 0.235 | 0.579 |  |
|  | Male (n=48) | 3.5 (2.75-4) |  |  |  |  |
| Optimization_Patient Management | Female (n=86) | 4 (3-5) | Z=1920 | 0.474 | 0.758 |  |
|  | Male (n=48) | 4.5 (4-5) |  |  |  |  |
| Optimization_Medical History Collection | Female (n=86) | 4 (3-5) | Z=2159.5 | 0.642 | 0.83 |  |
|  | Male (n=48) | 4 (3-5) |  |  |  |  |
| Optimization_Mental Status Examination | Female (n=86) | 4 (3-5) | Z=2324.5 | 0.213 | 0.579 |  |
|  | Male (n=48) | 4 (2-4.25) |  |  |  |  |
| Optimization_Medical Documentation Writing | Female (n=86) | 5 (4-5) | Z=1901 | 0.402 | 0.758 |  |
|  | Male (n=48) | 5 (4-5) |  |  |  |  |
| Optimization_Diagnostic Assistance | Female (n=86) | 4 (4-5) | Z=2310 | 0.226 | 0.579 |  |
|  | Male (n=48) | 4 (3-5) |  |  |  |  |
| Optimization_Treatment Planning and Outcome Prediction | Female (n=86) | 4 (4-5) | Z=2216.5 | 0.451 | 0.758 |  |
|  | Male (n=48) | 4 (3-5) |  |  |  |  |
| Optimization_Risk Assessment and Prognosis Estimation | Female (n=86) | 4 (4-5) | Z=2155 | 0.652 | 0.83 |  |
|  | Male (n=48) | 4 (3.75-5) |  |  |  |  |
| Optimization_Doctor-Patient Communication | Female (n=86) | 4 (3-5) | Z=2446 | 0.067 | 0.385 |  |
|  | Male (n=48) | 3.5 (2-4.25) |  |  |  |  |
| Optimization_Psychological Interventions | Female (n=86) | 4 (3-5) | Z=2458 | 0.059 | 0.385 |  |
|  | Male (n=48) | 3.5 (2.75-4.25) |  |  |  |  |

Table S2：Group differences between Age

| **Item** | **Group** | **Descriptive Statistics** | **Statistic** | **Raw P-value** | **P-value (FDR)** | **Post-hoc Test** |
| --- | --- | --- | --- | --- | --- | --- |
| AI Exposure & Learning | 18-23 (n=1) | 4 (4-4) | H=6.77 | 0.148 | 0.455 |  |
|  | 24-30 (n=45) | 3.16±1.05 |  |  |  |  |
|  | 31-40 (n=56) | 3 (2.25-4) |  |  |  |  |
|  | 41-50 (n=27) | 3.06±1.05 |  |  |  |  |
|  | >50 (n=5) | 2.33 (1.33-2.33) |  |  |  |  |
| AI Trust & Expectations | 18-23 (n=1) | 3.67 (3.67-3.67) | H=4.42 | 0.352 | 0.579 |  |
|  | 24-30 (n=45) | 3.39±0.86 |  |  |  |  |
|  | 31-40 (n=56) | 3.34±0.88 |  |  |  |  |
|  | 41-50 (n=27) | 3.4±0.77 |  |  |  |  |
|  | >50 (n=5) | 2.67 (2.33-3) |  |  |  |  |
| Needs_Clinical_Decision | 18-23 (n=1) | 5 (5-5) | H=8.1 | 0.088 | 0.379 |  |
|  | 24-30 (n=45) | 4.33 (3-5) |  |  |  |  |
|  | 31-40 (n=56) | 4 (3-4.33) |  |  |  |  |
|  | 41-50 (n=27) | 4.33 (4-4.83) |  |  |  |  |
|  | >50 (n=5) | 4 (3.67-4.33) |  |  |  |  |
| Needs_Routine_Tasks | 18-23 (n=1) | 4 (4-4) | H=1.72 | 0.787 | 0.84 |  |
|  | 24-30 (n=45) | 3.62±0.94 |  |  |  |  |
|  | 31-40 (n=56) | 3.75 (3-4.25) |  |  |  |  |
|  | 41-50 (n=27) | 3.73±0.81 |  |  |  |  |
|  | >50 (n=5) | 3.25 (2.5-4) |  |  |  |  |
| Needs_Interaction | 18-23 (n=1) | 3 (3-3) | H=3.14 | 0.535 | 0.651 |  |
|  | 24-30 (n=45) | 3 (2.5-4.5) |  |  |  |  |
|  | 31-40 (n=56) | 3 (2.38-4) |  |  |  |  |
|  | 41-50 (n=27) | 3.48±1.08 |  |  |  |  |
|  | >50 (n=5) | 3.5 (2.5-4) |  |  |  |  |
| Medical Needs | 18-23 (n=1) | 4.11 (4.11-4.11) | H=4.65 | 0.325 | 0.555 |  |
|  | 24-30 (n=45) | 3.68±0.88 |  |  |  |  |
|  | 31-40 (n=56) | 3.67 (2.89-4) |  |  |  |  |
|  | 41-50 (n=27) | 3.84±0.65 |  |  |  |  |
|  | >50 (n=5) | 3.56 (2.89-3.78) |  |  |  |  |
| Potential_Clinical_Efficiency | 18-23 (n=1) | 5 (5-5) | H=5.28 | 0.26 | 0.553 |  |
|  | 24-30 (n=45) | 4 (3.33-5) |  |  |  |  |
|  | 31-40 (n=56) | 4 (3.5-4.67) |  |  |  |  |
|  | 41-50 (n=27) | 4.5 (3.83-4.92) |  |  |  |  |
|  | >50 (n=5) | 4 (3.5-4.83) |  |  |  |  |
| Potential_Humanistic_Care | 18-23 (n=1) | 4.33 (4.33-4.33) | H=3.45 | 0.485 | 0.619 |  |
|  | 24-30 (n=45) | 3.67 (3-4.33) |  |  |  |  |
|  | 31-40 (n=56) | 3.33 (2.67-4) |  |  |  |  |
|  | 41-50 (n=27) | 3.67 (2.67-4.67) |  |  |  |  |
|  | >50 (n=5) | 3.67 (2.33-3.67) |  |  |  |  |
| Medical Potential | 18-23 (n=1) | 4.78 (4.78-4.78) | H=4.15 | 0.387 | 0.607 |  |
|  | 24-30 (n=45) | 3.83±0.91 |  |  |  |  |
|  | 31-40 (n=56) | 3.78 (3.19-4.36) |  |  |  |  |
|  | 41-50 (n=27) | 4.33 (3.5-4.83) |  |  |  |  |
|  | >50 (n=5) | 3.89 (2.89-4.44) |  |  |  |  |
| Optimization_Clinical_Efficiency | 18-23 (n=1) | 5 (5-5) | H=6.53 | 0.163 | 0.455 |  |
|  | 24-30 (n=45) | 4 (3.5-5) |  |  |  |  |
|  | 31-40 (n=56) | 4.08 (3.62-5) |  |  |  |  |
|  | 41-50 (n=27) | 4.83 (4.08-5) |  |  |  |  |
|  | >50 (n=5) | 3.67 (3.5-5) |  |  |  |  |
| Optimization_Humanistic_Care | 18-23 (n=1) | 5 (5-5) | H=6.6 | 0.159 | 0.455 |  |
|  | 24-30 (n=45) | 3.67 (3-4.33) |  |  |  |  |
|  | 31-40 (n=56) | 3.67 (2.33-4.67) |  |  |  |  |
|  | 41-50 (n=27) | 4.33 (3.33-5) |  |  |  |  |
|  | >50 (n=5) | 3.33 (2.33-3.33) |  |  |  |  |
| Medical Optimization | 18-23 (n=1) | 5 (5-5) | H=7.23 | 0.124 | 0.455 |  |
|  | 24-30 (n=45) | 4 (3.33-4.33) |  |  |  |  |
|  | 31-40 (n=56) | 4 (3.22-4.69) |  |  |  |  |
|  | 41-50 (n=27) | 4.56 (3.72-5) |  |  |  |  |
|  | >50 (n=5) | 3.44 (3.11-4.44) |  |  |  |  |
| Journals | 18-23 (n=1) | 1 (1-1) | H=8.93 | 0.063 | 0.323 |  |
|  | 24-30 (n=45) | 1 (0-1) |  |  |  |  |
|  | 31-40 (n=56) | 0 (0-1) |  |  |  |  |
|  | 41-50 (n=27) | 0 (0-1) |  |  |  |  |
|  | >50 (n=5) | 0 (0-0) |  |  |  |  |
| Online Courses | 18-23 (n=1) | 0 (0-0) | H=3.44 | 0.486 | 0.619 |  |
|  | 24-30 (n=45) | 0 (0-1) |  |  |  |  |
|  | 31-40 (n=56) | 0 (0-1) |  |  |  |  |
|  | 41-50 (n=27) | 0 (0-1) |  |  |  |  |
|  | >50 (n=5) | 0 (0-1) |  |  |  |  |
| Hospital Training | 18-23 (n=1) | 1 (1-1) | H=5.08 | 0.279 | 0.553 |  |
|  | 24-30 (n=45) | 0 (0-0) |  |  |  |  |
|  | 31-40 (n=56) | 0 (0-0) |  |  |  |  |
|  | 41-50 (n=27) | 0 (0-1) |  |  |  |  |
|  | >50 (n=5) | 0 (0-0) |  |  |  |  |
| Social Media | 18-23 (n=1) | 1 (1-1) | H=5.7 | 0.223 | 0.524 |  |
|  | 24-30 (n=45) | 1 (0-1) |  |  |  |  |
|  | 31-40 (n=56) | 1 (1-1) |  |  |  |  |
|  | 41-50 (n=27) | 1 (1-1) |  |  |  |  |
|  | >50 (n=5) | 1 (1-1) |  |  |  |  |
| Conferences | 18-23 (n=1) | 0 (0-0) | H=0.82 | 0.936 | 0.936 |  |
|  | 24-30 (n=45) | 0 (0-0) |  |  |  |  |
|  | 31-40 (n=56) | 0 (0-0) |  |  |  |  |
|  | 41-50 (n=27) | 0 (0-0) |  |  |  |  |
|  | >50 (n=5) | 0 (0-0) |  |  |  |  |
| Colleague Discussions | 18-23 (n=1) | 1 (1-1) | H=10.95 | 0.027 | 0.323 | 无显著两两差异 |
|  | 24-30 (n=45) | 0 (0-1) |  |  |  |  |
|  | 31-40 (n=56) | 1 (0-1) |  |  |  |  |
|  | 41-50 (n=27) | 1 (0.5-1) |  |  |  |  |
|  | >50 (n=5) | 0 (0-0) |  |  |  |  |
| News & Websites | 18-23 (n=1) | 1 (1-1) | H=10.34 | 0.035 | 0.323 | 无显著两两差异 |
|  | 24-30 (n=45) | 0 (0-1) |  |  |  |  |
|  | 31-40 (n=56) | 0 (0-1) |  |  |  |  |
|  | 41-50 (n=27) | 1 (0-1) |  |  |  |  |
|  | >50 (n=5) | 1 (1-1) |  |  |  |  |
| Guidelines | 18-23 (n=1) | 0 (0-0) | H=6.23 | 0.183 | 0.479 |  |
|  | 24-30 (n=45) | 0 (0-0) |  |  |  |  |
|  | 31-40 (n=56) | 0 (0-0) |  |  |  |  |
|  | 41-50 (n=27) | 0 (0-0) |  |  |  |  |
|  | >50 (n=5) | 0 (0-0) |  |  |  |  |
| Books | 18-23 (n=1) | 0 (0-0) | H=1.68 | 0.795 | 0.84 |  |
|  | 24-30 (n=45) | 0 (0-0) |  |  |  |  |
|  | 31-40 (n=56) | 0 (0-0) |  |  |  |  |
|  | 41-50 (n=27) | 0 (0-0) |  |  |  |  |
|  | >50 (n=5) | 0 (0-0) |  |  |  |  |
| Vendor Docs | 18-23 (n=1) | 1 (1-1) | H=8.91 | 0.064 | 0.323 |  |
|  | 24-30 (n=45) | 0 (0-0) |  |  |  |  |
|  | 31-40 (n=56) | 0 (0-0) |  |  |  |  |
|  | 41-50 (n=27) | 0 (0-0) |  |  |  |  |
|  | >50 (n=5) | 0 (0-0) |  |  |  |  |
| Other | 18-23 (n=1) | 0 (0-0) | H=7.84 | 0.098 | 0.39 |  |
|  | 24-30 (n=45) | 0 (0-0) |  |  |  |  |
|  | 31-40 (n=56) | 0 (0-0) |  |  |  |  |
|  | 41-50 (n=27) | 0 (0-0) |  |  |  |  |
|  | >50 (n=5) | 0 (0-0) |  |  |  |  |
| Understanding | 18-23 (n=1) | 2 (2-2) | H=6.15 | 0.188 | 0.479 |  |
|  | 24-30 (n=45) | 3 (3-4) |  |  |  |  |
|  | 31-40 (n=56) | 3 (2-3) |  |  |  |  |
|  | 41-50 (n=27) | 3 (2-3) |  |  |  |  |
|  | >50 (n=5) | 2 (2-3) |  |  |  |  |
| Reading Articles | 18-23 (n=1) | 3 (3-3) | H=2.4 | 0.662 | 0.773 |  |
|  | 24-30 (n=45) | 3 (2-3) |  |  |  |  |
|  | 31-40 (n=56) | 2 (2-3.25) |  |  |  |  |
|  | 41-50 (n=27) | 2 (1.5-3) |  |  |  |  |
|  | >50 (n=5) | 2 (1-2) |  |  |  |  |
| Effective Use | 18-23 (n=1) | 4 (4-4) | H=9.46 | 0.051 | 0.323 |  |
|  | 24-30 (n=45) | 3 (3-4) |  |  |  |  |
|  | 31-40 (n=56) | 3 (2-4) |  |  |  |  |
|  | 41-50 (n=27) | 3 (2-4) |  |  |  |  |
|  | >50 (n=5) | 2 (1-2) |  |  |  |  |
| Access & Use | 18-23 (n=1) | 5 (5-5) | H=11.87 | 0.018 | 0.323 | >50 vs 24-30 |
|  | 24-30 (n=45) | 4 (3-4) |  |  |  |  |
|  | 31-40 (n=56) | 3 (2-4) |  |  |  |  |
|  | 41-50 (n=27) | 4 (2-4) |  |  |  |  |
|  | >50 (n=5) | 2 (1-2) |  |  |  |  |
| Active Seeking | 18-23 (n=1) | 4 (4-4) | H=3.74 | 0.442 | 0.614 |  |
|  | 24-30 (n=45) | 3 (3-4) |  |  |  |  |
|  | 31-40 (n=56) | 4 (2-4) |  |  |  |  |
|  | 41-50 (n=27) | 4 (3-4) |  |  |  |  |
|  | >50 (n=5) | 3 (2-3) |  |  |  |  |
| Future Importance | 18-23 (n=1) | 5 (5-5) | H=10.82 | 0.029 | 0.323 | 41-50 vs 24-30 |
|  | 24-30 (n=45) | 4 (3-4) |  |  |  |  |
|  | 31-40 (n=56) | 4 (3-5) |  |  |  |  |
|  | 41-50 (n=27) | 5 (4-5) |  |  |  |  |
|  | >50 (n=5) | 4 (4-4) |  |  |  |  |
| Needs_Patient Management | 18-23 (n=1) | 3 (3-3) | H=3.96 | 0.412 | 0.607 |  |
|  | 24-30 (n=45) | 4 (3-5) |  |  |  |  |
|  | 31-40 (n=56) | 4 (3-4) |  |  |  |  |
|  | 41-50 (n=27) | 4 (4-5) |  |  |  |  |
|  | >50 (n=5) | 4 (4-4) |  |  |  |  |
| Needs_Medical History Collection | 18-23 (n=1) | 5 (5-5) | H=2.56 | 0.633 | 0.755 |  |
|  | 24-30 (n=45) | 3 (3-4) |  |  |  |  |
|  | 31-40 (n=56) | 3.5 (2.75-4) |  |  |  |  |
|  | 41-50 (n=27) | 4 (3-4) |  |  |  |  |
|  | >50 (n=5) | 3 (2-4) |  |  |  |  |
| Needs_Mental Status Examination | 18-23 (n=1) | 3 (3-3) | H=1.44 | 0.837 | 0.868 |  |
|  | 24-30 (n=45) | 3 (2-4) |  |  |  |  |
|  | 31-40 (n=56) | 3 (2-4) |  |  |  |  |
|  | 41-50 (n=27) | 3 (2-4) |  |  |  |  |
|  | >50 (n=5) | 2 (1-3) |  |  |  |  |
| Needs_Medical Documentation Writing | 18-23 (n=1) | 5 (5-5) | H=5.05 | 0.282 | 0.553 |  |
|  | 24-30 (n=45) | 4 (4-5) |  |  |  |  |
|  | 31-40 (n=56) | 4 (3-5) |  |  |  |  |
|  | 41-50 (n=27) | 5 (4-5) |  |  |  |  |
|  | >50 (n=5) | 4 (4-5) |  |  |  |  |
| Needs_Diagnostic Assistance | 18-23 (n=1) | 5 (5-5) | H=8.45 | 0.076 | 0.356 |  |
|  | 24-30 (n=45) | 4 (3-5) |  |  |  |  |
|  | 31-40 (n=56) | 4 (3-4) |  |  |  |  |
|  | 41-50 (n=27) | 4 (4-5) |  |  |  |  |
|  | >50 (n=5) | 4 (3-5) |  |  |  |  |
| Needs_Treatment Planning and Outcome Prediction | 18-23 (n=1) | 5 (5-5) | H=4.89 | 0.299 | 0.553 |  |
|  | 24-30 (n=45) | 4 (3-5) |  |  |  |  |
|  | 31-40 (n=56) | 4 (3-4.25) |  |  |  |  |
|  | 41-50 (n=27) | 4 (4-5) |  |  |  |  |
|  | >50 (n=5) | 4 (4-4) |  |  |  |  |
| Needs_Risk Assessment and Prognosis Estimation | 18-23 (n=1) | 5 (5-5) | H=6.92 | 0.14 | 0.455 |  |
|  | 24-30 (n=45) | 4 (3-5) |  |  |  |  |
|  | 31-40 (n=56) | 4 (3-4) |  |  |  |  |
|  | 41-50 (n=27) | 4 (4-5) |  |  |  |  |
|  | >50 (n=5) | 4 (4-4) |  |  |  |  |
| Needs_Doctor-Patient Communication | 18-23 (n=1) | 3 (3-3) | H=2.23 | 0.694 | 0.793 |  |
|  | 24-30 (n=45) | 3 (2-5) |  |  |  |  |
|  | 31-40 (n=56) | 3 (2-4) |  |  |  |  |
|  | 41-50 (n=27) | 4 (2.5-4) |  |  |  |  |
|  | >50 (n=5) | 3 (3-4) |  |  |  |  |
| Needs_Psychological Interventions | 18-23 (n=1) | 3 (3-3) | H=3.72 | 0.445 | 0.614 |  |
|  | 24-30 (n=45) | 3 (2-5) |  |  |  |  |
|  | 31-40 (n=56) | 3 (2-4) |  |  |  |  |
|  | 41-50 (n=27) | 4 (3-4.5) |  |  |  |  |
|  | >50 (n=5) | 4 (2-4) |  |  |  |  |
| Potential_Patient Management | 18-23 (n=1) | 5 (5-5) | H=5.68 | 0.225 | 0.524 |  |
|  | 24-30 (n=45) | 4 (3-5) |  |  |  |  |
|  | 31-40 (n=56) | 4 (3-5) |  |  |  |  |
|  | 41-50 (n=27) | 5 (4-5) |  |  |  |  |
|  | >50 (n=5) | 4 (4-5) |  |  |  |  |
| Potential_Medical History Collection | 18-23 (n=1) | 5 (5-5) | H=2.06 | 0.725 | 0.805 |  |
|  | 24-30 (n=45) | 4 (3-5) |  |  |  |  |
|  | 31-40 (n=56) | 4 (3-5) |  |  |  |  |
|  | 41-50 (n=27) | 4 (3-5) |  |  |  |  |
|  | >50 (n=5) | 4 (2-5) |  |  |  |  |
| Potential_Mental Status Examination | 18-23 (n=1) | 5 (5-5) | H=4.05 | 0.4 | 0.607 |  |
|  | 24-30 (n=45) | 4 (3-5) |  |  |  |  |
|  | 31-40 (n=56) | 3 (2-4) |  |  |  |  |
|  | 41-50 (n=27) | 3 (3-5) |  |  |  |  |
|  | >50 (n=5) | 3 (1-4) |  |  |  |  |
| Potential_Medical Documentation Writing | 18-23 (n=1) | 5 (5-5) | H=3.55 | 0.47 | 0.619 |  |
|  | 24-30 (n=45) | 5 (4-5) |  |  |  |  |
|  | 31-40 (n=56) | 4 (3.75-5) |  |  |  |  |
|  | 41-50 (n=27) | 5 (4-5) |  |  |  |  |
|  | >50 (n=5) | 4 (4-5) |  |  |  |  |
| Potential_Diagnostic Assistance | 18-23 (n=1) | 5 (5-5) | H=11.41 | 0.022 | 0.323 | 41-50 vs 31-40 |
|  | 24-30 (n=45) | 4 (3-5) |  |  |  |  |
|  | 31-40 (n=56) | 4 (3.75-5) |  |  |  |  |
|  | 41-50 (n=27) | 5 (4-5) |  |  |  |  |
|  | >50 (n=5) | 4 (4-5) |  |  |  |  |
| Potential_Treatment Planning and Outcome Prediction | 18-23 (n=1) | 5 (5-5) | H=4.82 | 0.306 | 0.553 |  |
|  | 24-30 (n=45) | 4 (3-5) |  |  |  |  |
|  | 31-40 (n=56) | 4 (4-5) |  |  |  |  |
|  | 41-50 (n=27) | 5 (4-5) |  |  |  |  |
|  | >50 (n=5) | 4 (3-4) |  |  |  |  |
| Potential_Risk Assessment and Prognosis Estimation | 18-23 (n=1) | 5 (5-5) | H=4.9 | 0.298 | 0.553 |  |
|  | 24-30 (n=45) | 4 (3-5) |  |  |  |  |
|  | 31-40 (n=56) | 4 (4-5) |  |  |  |  |
|  | 41-50 (n=27) | 5 (4-5) |  |  |  |  |
|  | >50 (n=5) | 4 (4-5) |  |  |  |  |
| Potential_Doctor-Patient Communication | 18-23 (n=1) | 4 (4-4) | H=2.02 | 0.733 | 0.805 |  |
|  | 24-30 (n=45) | 3 (3-5) |  |  |  |  |
|  | 31-40 (n=56) | 3 (2-4) |  |  |  |  |
|  | 41-50 (n=27) | 4 (3-5) |  |  |  |  |
|  | >50 (n=5) | 4 (3-4) |  |  |  |  |
| Potential_Psychological Interventions | 18-23 (n=1) | 4 (4-4) | H=3.69 | 0.449 | 0.614 |  |
|  | 24-30 (n=45) | 3 (3-5) |  |  |  |  |
|  | 31-40 (n=56) | 4 (2-4) |  |  |  |  |
|  | 41-50 (n=27) | 4 (3-5) |  |  |  |  |
|  | >50 (n=5) | 3 (3-4) |  |  |  |  |
| Optimization_Patient Management | 18-23 (n=1) | 5 (5-5) | H=1.27 | 0.866 | 0.882 |  |
|  | 24-30 (n=45) | 4 (4-5) |  |  |  |  |
|  | 31-40 (n=56) | 4 (3-5) |  |  |  |  |
|  | 41-50 (n=27) | 5 (4-5) |  |  |  |  |
|  | >50 (n=5) | 4 (4-5) |  |  |  |  |
| Optimization_Medical History Collection | 18-23 (n=1) | 5 (5-5) | H=3.31 | 0.507 | 0.631 |  |
|  | 24-30 (n=45) | 4 (3-5) |  |  |  |  |
|  | 31-40 (n=56) | 4 (3-5) |  |  |  |  |
|  | 41-50 (n=27) | 4 (3.5-5) |  |  |  |  |
|  | >50 (n=5) | 3 (2-5) |  |  |  |  |
| Optimization_Mental Status Examination | 18-23 (n=1) | 5 (5-5) | H=4.63 | 0.327 | 0.555 |  |
|  | 24-30 (n=45) | 4 (3-5) |  |  |  |  |
|  | 31-40 (n=56) | 4 (2-5) |  |  |  |  |
|  | 41-50 (n=27) | 4 (3-5) |  |  |  |  |
|  | >50 (n=5) | 2 (2-4) |  |  |  |  |
| Optimization_Medical Documentation Writing | 18-23 (n=1) | 5 (5-5) | H=5.08 | 0.279 | 0.553 |  |
|  | 24-30 (n=45) | 5 (4-5) |  |  |  |  |
|  | 31-40 (n=56) | 4 (4-5) |  |  |  |  |
|  | 41-50 (n=27) | 5 (4.5-5) |  |  |  |  |
|  | >50 (n=5) | 4 (4-5) |  |  |  |  |
| Optimization_Diagnostic Assistance | 18-23 (n=1) | 5 (5-5) | H=9.17 | 0.057 | 0.323 |  |
|  | 24-30 (n=45) | 4 (3-5) |  |  |  |  |
|  | 31-40 (n=56) | 4 (3-5) |  |  |  |  |
|  | 41-50 (n=27) | 5 (4-5) |  |  |  |  |
|  | >50 (n=5) | 4 (3-5) |  |  |  |  |
| Optimization_Treatment Planning and Outcome Prediction | 18-23 (n=1) | 5 (5-5) | H=9.01 | 0.061 | 0.323 |  |
|  | 24-30 (n=45) | 4 (3-5) |  |  |  |  |
|  | 31-40 (n=56) | 4 (3.75-5) |  |  |  |  |
|  | 41-50 (n=27) | 5 (4-5) |  |  |  |  |
|  | >50 (n=5) | 4 (4-5) |  |  |  |  |
| Optimization_Risk Assessment and Prognosis Estimation | 18-23 (n=1) | 5 (5-5) | H=10.8 | 0.029 | 0.323 | 41-50 vs 24-30 |
|  | 24-30 (n=45) | 4 (3-5) |  |  |  |  |
|  | 31-40 (n=56) | 4 (3.75-5) |  |  |  |  |
|  | 41-50 (n=27) | 5 (4-5) |  |  |  |  |
|  | >50 (n=5) | 4 (3-5) |  |  |  |  |
| Optimization_Doctor-Patient Communication | 18-23 (n=1) | 5 (5-5) | H=4.01 | 0.405 | 0.607 |  |
|  | 24-30 (n=45) | 4 (3-5) |  |  |  |  |
|  | 31-40 (n=56) | 4 (2-5) |  |  |  |  |
|  | 41-50 (n=27) | 4 (3.5-5) |  |  |  |  |
|  | >50 (n=5) | 3 (3-4) |  |  |  |  |
| Optimization_Psychological Interventions | 18-23 (n=1) | 5 (5-5) | H=6.72 | 0.151 | 0.455 |  |
|  | 24-30 (n=45) | 4 (3-5) |  |  |  |  |
|  | 31-40 (n=56) | 4 (2-5) |  |  |  |  |
|  | 41-50 (n=27) | 5 (3.5-5) |  |  |  |  |
|  | >50 (n=5) | 3 (3-4) |  |  |  |  |

Table S3：Group differences among education background

| **Item** | **Group** | **Descriptive Statistics** | **Statistic** | **Raw P-value** | **P-value (FDR)** | **Post-hoc Test** |
| --- | --- | --- | --- | --- | --- | --- |
| AI Exposure & Learning | College (n=4) | 2.67 (1.83-3.67) | H=5.82 | 0.121 | 0.856 |  |
|  | Bachelor (n=51) | 2.82±0.96 |  |  |  |  |
|  | Master (n=62) | 3 (2.33-4) |  |  |  |  |
|  | PhD (n=17) | 3.55±1.17 |  |  |  |  |
| AI Trust & Expectations | College (n=4) | 3.17 (2.08-4) | H=4.09 | 0.251 | 0.856 |  |
|  | Bachelor (n=51) | 3.19±0.88 |  |  |  |  |
|  | Master (n=62) | 3.33 (3-3.92) |  |  |  |  |
|  | PhD (n=17) | 4 (3-4) |  |  |  |  |
| Needs_Clinical_Decision | College (n=4) | 3.33 (2.83-3.75) | H=3.69 | 0.297 | 0.856 |  |
|  | Bachelor (n=51) | 4 (3.17-4.67) |  |  |  |  |
|  | Master (n=62) | 4 (3.33-5) |  |  |  |  |
|  | PhD (n=17) | 4.67 (3.67-4.67) |  |  |  |  |
| Needs_Routine_Tasks | College (n=4) | 3.5 (3.12-3.62) | H=1.94 | 0.584 | 0.901 |  |
|  | Bachelor (n=51) | 3.75 (2.88-4.12) |  |  |  |  |
|  | Master (n=62) | 3.67±0.87 |  |  |  |  |
|  | PhD (n=17) | 3.6±1 |  |  |  |  |
| Needs_Interaction | College (n=4) | 3.25 (2.5-3.62) | H=1.92 | 0.588 | 0.901 |  |
|  | Bachelor (n=51) | 3.5 (2.5-4) |  |  |  |  |
|  | Master (n=62) | 3.5 (2.5-4) |  |  |  |  |
|  | PhD (n=17) | 2.88±1.17 |  |  |  |  |
| Medical Needs | College (n=4) | 3.11 (2.86-3.42) | H=1.97 | 0.579 | 0.901 |  |
|  | Bachelor (n=51) | 3.54±0.94 |  |  |  |  |
|  | Master (n=62) | 3.7±0.77 |  |  |  |  |
|  | PhD (n=17) | 3.78 (3.44-4) |  |  |  |  |
| Potential_Clinical_Efficiency | College (n=4) | 4 (3.5-4.25) | H=0.2 | 0.978 | 0.981 |  |
|  | Bachelor (n=51) | 4 (3.42-5) |  |  |  |  |
|  | Master (n=62) | 4.08 (3.38-4.79) |  |  |  |  |
|  | PhD (n=17) | 4 (3.83-4.67) |  |  |  |  |
| Potential_Humanistic_Care | College (n=4) | 3 (2.33-3.75) | H=2.29 | 0.514 | 0.901 |  |
|  | Bachelor (n=51) | 3.67 (2.67-5) |  |  |  |  |
|  | Master (n=62) | 3.5 (2.67-4.33) |  |  |  |  |
|  | PhD (n=17) | 3.2±1.05 |  |  |  |  |
| Medical Potential | College (n=4) | 3.94 (3.44-4.03) | H=0.52 | 0.913 | 0.97 |  |
|  | Bachelor (n=51) | 4 (3.22-4.89) |  |  |  |  |
|  | Master (n=62) | 3.83 (3.22-4.56) |  |  |  |  |
|  | PhD (n=17) | 3.78 (3.56-4.11) |  |  |  |  |
| Optimization_Clinical_Efficiency | College (n=4) | 4 (3.5-4.17) | H=1.42 | 0.701 | 0.901 |  |
|  | Bachelor (n=51) | 4.17 (3.5-5) |  |  |  |  |
|  | Master (n=62) | 4.17 (3.67-5) |  |  |  |  |
|  | PhD (n=17) | 4.16±0.79 |  |  |  |  |
| Optimization_Humanistic_Care | College (n=4) | 3 (2.17-3.75) | H=2.3 | 0.512 | 0.901 |  |
|  | Bachelor (n=51) | 4 (2.33-4.83) |  |  |  |  |
|  | Master (n=62) | 3.67 (3-5) |  |  |  |  |
|  | PhD (n=17) | 4 (2.67-4.67) |  |  |  |  |
| Medical Optimization | College (n=4) | 3.78 (3.28-3.92) | H=2.01 | 0.569 | 0.901 |  |
|  | Bachelor (n=51) | 4 (3.11-4.89) |  |  |  |  |
|  | Master (n=62) | 4 (3.47-5) |  |  |  |  |
|  | PhD (n=17) | 3.98±0.84 |  |  |  |  |
| Journals | College (n=4) | 0 (0-0) | H=2.66 | 0.448 | 0.901 |  |
|  | Bachelor (n=51) | 0 (0-1) |  |  |  |  |
|  | Master (n=62) | 0 (0-1) |  |  |  |  |
|  | PhD (n=17) | 0 (0-1) |  |  |  |  |
| Online Courses | College (n=4) | 0 (0-0) | H=5.64 | 0.13 | 0.856 |  |
|  | Bachelor (n=51) | 0 (0-1) |  |  |  |  |
|  | Master (n=62) | 0 (0-1) |  |  |  |  |
|  | PhD (n=17) | 0 (0-1) |  |  |  |  |
| Hospital Training | College (n=4) | 0.5 (0-1) | H=3.64 | 0.303 | 0.856 |  |
|  | Bachelor (n=51) | 0 (0-1) |  |  |  |  |
|  | Master (n=62) | 0 (0-0) |  |  |  |  |
|  | PhD (n=17) | 0 (0-0) |  |  |  |  |
| Social Media | College (n=4) | 1 (0.75-1) | H=1.55 | 0.67 | 0.901 |  |
|  | Bachelor (n=51) | 1 (1-1) |  |  |  |  |
|  | Master (n=62) | 1 (1-1) |  |  |  |  |
|  | PhD (n=17) | 1 (1-1) |  |  |  |  |
| Conferences | College (n=4) | 0 (0-0) | H=7.57 | 0.056 | 0.856 |  |
|  | Bachelor (n=51) | 0 (0-0.5) |  |  |  |  |
|  | Master (n=62) | 0 (0-0) |  |  |  |  |
|  | PhD (n=17) | 0 (0-0) |  |  |  |  |
| Colleague Discussions | College (n=4) | 1 (0.75-1) | H=1.17 | 0.761 | 0.908 |  |
|  | Bachelor (n=51) | 1 (0-1) |  |  |  |  |
|  | Master (n=62) | 1 (0-1) |  |  |  |  |
|  | PhD (n=17) | 1 (0-1) |  |  |  |  |
| News & Websites | College (n=4) | 0.5 (0-1) | H=5.7 | 0.127 | 0.856 |  |
|  | Bachelor (n=51) | 1 (0-1) |  |  |  |  |
|  | Master (n=62) | 1 (0-1) |  |  |  |  |
|  | PhD (n=17) | 0 (0-1) |  |  |  |  |
| Guidelines | College (n=4) | 0 (0-0) | H=2.7 | 0.441 | 0.901 |  |
|  | Bachelor (n=51) | 0 (0-0) |  |  |  |  |
|  | Master (n=62) | 0 (0-0) |  |  |  |  |
|  | PhD (n=17) | 0 (0-0) |  |  |  |  |
| Books | College (n=4) | 0 (0-0.25) | H=1.58 | 0.663 | 0.901 |  |
|  | Bachelor (n=51) | 0 (0-0) |  |  |  |  |
|  | Master (n=62) | 0 (0-0) |  |  |  |  |
|  | PhD (n=17) | 0 (0-1) |  |  |  |  |
| Vendor Docs | College (n=4) | 0 (0-0) | H=5.06 | 0.167 | 0.856 |  |
|  | Bachelor (n=51) | 0 (0-0) |  |  |  |  |
|  | Master (n=62) | 0 (0-0) |  |  |  |  |
|  | PhD (n=17) | 0 (0-0) |  |  |  |  |
| Other | College (n=4) | 0 (0-0) | H=4.96 | 0.175 | 0.856 |  |
|  | Bachelor (n=51) | 0 (0-0) |  |  |  |  |
|  | Master (n=62) | 0 (0-0) |  |  |  |  |
|  | PhD (n=17) | 0 (0-0) |  |  |  |  |
| Understanding | College (n=4) | 3 (1.75-4) | H=2.67 | 0.445 | 0.901 |  |
|  | Bachelor (n=51) | 3 (2-3) |  |  |  |  |
|  | Master (n=62) | 3 (2.25-3.75) |  |  |  |  |
|  | PhD (n=17) | 3 (2-4) |  |  |  |  |
| Reading Articles | College (n=4) | 2.5 (1.75-3.25) | H=5.1 | 0.164 | 0.856 |  |
|  | Bachelor (n=51) | 2 (1.5-3) |  |  |  |  |
|  | Master (n=62) | 2.5 (2-3.75) |  |  |  |  |
|  | PhD (n=17) | 3.06±1.3 |  |  |  |  |
| Effective Use | College (n=4) | 3 (1.75-4) | H=2.55 | 0.466 | 0.901 |  |
|  | Bachelor (n=51) | 3 (2-4) |  |  |  |  |
|  | Master (n=62) | 3 (2-4) |  |  |  |  |
|  | PhD (n=17) | 3.47±1.12 |  |  |  |  |
| Access & Use | College (n=4) | 3 (1.75-4.25) | H=4.83 | 0.184 | 0.856 |  |
|  | Bachelor (n=51) | 3 (2-4) |  |  |  |  |
|  | Master (n=62) | 3 (2-4) |  |  |  |  |
|  | PhD (n=17) | 4 (3-5) |  |  |  |  |
| Active Seeking | College (n=4) | 2.5 (2-3.5) | H=7 | 0.072 | 0.856 |  |
|  | Bachelor (n=51) | 3 (3-4) |  |  |  |  |
|  | Master (n=62) | 3 (3-4) |  |  |  |  |
|  | PhD (n=17) | 4 (4-5) |  |  |  |  |
| Future Importance | College (n=4) | 3.5 (2.75-4) | H=5.15 | 0.161 | 0.856 |  |
|  | Bachelor (n=51) | 4 (3-5) |  |  |  |  |
|  | Master (n=62) | 4 (3-5) |  |  |  |  |
|  | PhD (n=17) | 4 (4-5) |  |  |  |  |
| Needs_Patient Management | College (n=4) | 4 (3.5-4.25) | H=1.45 | 0.694 | 0.901 |  |
|  | Bachelor (n=51) | 4 (3-5) |  |  |  |  |
|  | Master (n=62) | 4 (3-5) |  |  |  |  |
|  | PhD (n=17) | 4 (3-4) |  |  |  |  |
| Needs_Medical History Collection | College (n=4) | 3.5 (2.5-4.25) | H=1.32 | 0.724 | 0.901 |  |
|  | Bachelor (n=51) | 3 (2.5-4) |  |  |  |  |
|  | Master (n=62) | 4 (3-4) |  |  |  |  |
|  | PhD (n=17) | 3.53±1.18 |  |  |  |  |
| Needs_Mental Status Examination | College (n=4) | 2.5 (1.75-3.25) | H=0.67 | 0.88 | 0.97 |  |
|  | Bachelor (n=51) | 3 (2-4) |  |  |  |  |
|  | Master (n=62) | 3 (2-4) |  |  |  |  |
|  | PhD (n=17) | 3±1.32 |  |  |  |  |
| Needs_Medical Documentation Writing | College (n=4) | 3.5 (3-4) | H=6.44 | 0.092 | 0.856 |  |
|  | Bachelor (n=51) | 4 (3-5) |  |  |  |  |
|  | Master (n=62) | 4 (4-5) |  |  |  |  |
|  | PhD (n=17) | 5 (4-5) |  |  |  |  |
| Needs_Diagnostic Assistance | College (n=4) | 3 (2.75-3.25) | H=5.43 | 0.143 | 0.856 |  |
|  | Bachelor (n=51) | 4 (3-5) |  |  |  |  |
|  | Master (n=62) | 4 (4-5) |  |  |  |  |
|  | PhD (n=17) | 4 (4-5) |  |  |  |  |
| Needs_Treatment Planning and Outcome Prediction | College (n=4) | 3.5 (3-4) | H=3.93 | 0.269 | 0.856 |  |
|  | Bachelor (n=51) | 4 (3.5-5) |  |  |  |  |
|  | Master (n=62) | 4 (3.25-5) |  |  |  |  |
|  | PhD (n=17) | 5 (4-5) |  |  |  |  |
| Needs_Risk Assessment and Prognosis Estimation | College (n=4) | 3.5 (2.75-4) | H=2.92 | 0.404 | 0.901 |  |
|  | Bachelor (n=51) | 4 (3-5) |  |  |  |  |
|  | Master (n=62) | 4 (3-5) |  |  |  |  |
|  | PhD (n=17) | 4 (4-5) |  |  |  |  |
| Needs_Doctor-Patient Communication | College (n=4) | 3 (2.5-3.25) | H=1.72 | 0.633 | 0.901 |  |
|  | Bachelor (n=51) | 3 (2-4) |  |  |  |  |
|  | Master (n=62) | 3 (2.25-4) |  |  |  |  |
|  | PhD (n=17) | 2.88±1.36 |  |  |  |  |
| Needs_Psychological Interventions | College (n=4) | 3.5 (2.5-4) | H=1.92 | 0.59 | 0.901 |  |
|  | Bachelor (n=51) | 3 (3-4) |  |  |  |  |
|  | Master (n=62) | 3 (2-4) |  |  |  |  |
|  | PhD (n=17) | 3 (2-4) |  |  |  |  |
| Potential_Patient Management | College (n=4) | 4 (3.75-4.25) | H=2.81 | 0.421 | 0.901 |  |
|  | Bachelor (n=51) | 4 (3-5) |  |  |  |  |
|  | Master (n=62) | 4 (4-5) |  |  |  |  |
|  | PhD (n=17) | 4 (3-5) |  |  |  |  |
| Potential_Medical History Collection | College (n=4) | 4 (3.25-4.25) | H=0.18 | 0.981 | 0.981 |  |
|  | Bachelor (n=51) | 4 (3-5) |  |  |  |  |
|  | Master (n=62) | 4 (3-5) |  |  |  |  |
|  | PhD (n=17) | 4 (3-5) |  |  |  |  |
| Potential_Mental Status Examination | College (n=4) | 3.5 (2.5-4) | H=0.38 | 0.945 | 0.98 |  |
|  | Bachelor (n=51) | 3 (2.5-5) |  |  |  |  |
|  | Master (n=62) | 3 (3-4.75) |  |  |  |  |
|  | PhD (n=17) | 3.35±1.22 |  |  |  |  |
| Potential_Medical Documentation Writing | College (n=4) | 4 (3.75-4.25) | H=1.38 | 0.709 | 0.901 |  |
|  | Bachelor (n=51) | 5 (4-5) |  |  |  |  |
|  | Master (n=62) | 5 (4-5) |  |  |  |  |
|  | PhD (n=17) | 5 (4-5) |  |  |  |  |
| Potential_Diagnostic Assistance | College (n=4) | 4 (3.25-4.25) | H=0.63 | 0.89 | 0.97 |  |
|  | Bachelor (n=51) | 4 (3-5) |  |  |  |  |
|  | Master (n=62) | 4 (4-5) |  |  |  |  |
|  | PhD (n=17) | 4 (4-5) |  |  |  |  |
| Potential_Treatment Planning and Outcome Prediction | College (n=4) | 4 (3.5-4.25) | H=0.5 | 0.918 | 0.97 |  |
|  | Bachelor (n=51) | 4 (3-5) |  |  |  |  |
|  | Master (n=62) | 4 (3-5) |  |  |  |  |
|  | PhD (n=17) | 4 (4-5) |  |  |  |  |
| Potential_Risk Assessment and Prognosis Estimation | College (n=4) | 4 (3.5-4.25) | H=0.94 | 0.815 | 0.951 |  |
|  | Bachelor (n=51) | 4 (4-5) |  |  |  |  |
|  | Master (n=62) | 4 (3.25-5) |  |  |  |  |
|  | PhD (n=17) | 4 (4-5) |  |  |  |  |
| Potential_Doctor-Patient Communication | College (n=4) | 3.5 (2.75-4) | H=1.92 | 0.589 | 0.901 |  |
|  | Bachelor (n=51) | 4 (2.5-5) |  |  |  |  |
|  | Master (n=62) | 3.5 (2.25-5) |  |  |  |  |
|  | PhD (n=17) | 3 (3-4) |  |  |  |  |
| Potential_Psychological Interventions | College (n=4) | 3 (2.75-3.25) | H=3.86 | 0.277 | 0.856 |  |
|  | Bachelor (n=51) | 4 (3-5) |  |  |  |  |
|  | Master (n=62) | 4 (3-4.75) |  |  |  |  |
|  | PhD (n=17) | 3 (3-4) |  |  |  |  |
| Optimization_Patient Management | College (n=4) | 4 (3.75-4.25) | H=1.46 | 0.692 | 0.901 |  |
|  | Bachelor (n=51) | 5 (3-5) |  |  |  |  |
|  | Master (n=62) | 4.5 (4-5) |  |  |  |  |
|  | PhD (n=17) | 4 (3-5) |  |  |  |  |
| Optimization_Medical History Collection | College (n=4) | 4 (3.25-4.25) | H=0.78 | 0.853 | 0.97 |  |
|  | Bachelor (n=51) | 4 (3-5) |  |  |  |  |
|  | Master (n=62) | 4 (3-5) |  |  |  |  |
|  | PhD (n=17) | 4 (3-5) |  |  |  |  |
| Optimization_Mental Status Examination | College (n=4) | 2.5 (1-4) | H=3.74 | 0.291 | 0.856 |  |
|  | Bachelor (n=51) | 4 (2-5) |  |  |  |  |
|  | Master (n=62) | 4 (3-5) |  |  |  |  |
|  | PhD (n=17) | 4 (2-5) |  |  |  |  |
| Optimization_Medical Documentation Writing | College (n=4) | 4 (3.75-4.25) | H=4.35 | 0.226 | 0.856 |  |
|  | Bachelor (n=51) | 4 (3.5-5) |  |  |  |  |
|  | Master (n=62) | 5 (4-5) |  |  |  |  |
|  | PhD (n=17) | 5 (4-5) |  |  |  |  |
| Optimization_Diagnostic Assistance | College (n=4) | 4 (3.25-4) | H=3.62 | 0.306 | 0.856 |  |
|  | Bachelor (n=51) | 4 (3-5) |  |  |  |  |
|  | Master (n=62) | 4 (4-5) |  |  |  |  |
|  | PhD (n=17) | 4 (4-5) |  |  |  |  |
| Optimization_Treatment Planning and Outcome Prediction | College (n=4) | 4 (3.5-4) | H=2.29 | 0.515 | 0.901 |  |
|  | Bachelor (n=51) | 4 (3-5) |  |  |  |  |
|  | Master (n=62) | 4 (4-5) |  |  |  |  |
|  | PhD (n=17) | 4 (4-5) |  |  |  |  |
| Optimization_Risk Assessment and Prognosis Estimation | College (n=4) | 4 (3.5-4.25) | H=1.16 | 0.762 | 0.908 |  |
|  | Bachelor (n=51) | 4 (3-5) |  |  |  |  |
|  | Master (n=62) | 4 (4-5) |  |  |  |  |
|  | PhD (n=17) | 4 (4-5) |  |  |  |  |
| Optimization_Doctor-Patient Communication | College (n=4) | 3.5 (2.75-4) | H=2.03 | 0.565 | 0.901 |  |
|  | Bachelor (n=51) | 4 (2-5) |  |  |  |  |
|  | Master (n=62) | 4 (3-5) |  |  |  |  |
|  | PhD (n=17) | 4 (3-5) |  |  |  |  |
| Optimization_Psychological Interventions | College (n=4) | 3 (2.75-3.25) | H=1.77 | 0.622 | 0.901 |  |
|  | Bachelor (n=51) | 4 (3-5) |  |  |  |  |
|  | Master (n=62) | 4 (3-5) |  |  |  |  |
|  | PhD (n=17) | 4 (3-5) |  |  |  |  |

Table S4：Group differences among hospital level

| **Item** | **Group** | **Descriptive Statistics** | **Statistic** | **Raw P-value** | **P-value (FDR)** | **Post-hoc Test** |
| --- | --- | --- | --- | --- | --- | --- |
| AI Exposure & Learning | Primary Hospital (n=13) | 2.33 (2-2.33) | H=6.09 | 0.048 | 0.918 | Teritary HospitalA vs Primary Hospital |
|  | Secondary Hospital (n=27) | 2.95±1.11 |  |  |  |  |
|  | Teritary HospitalA (n=94) | 3.15±1.04 |  |  |  |  |
| AI Trust & Expectations | Primary Hospital (n=13) | 2.97±1 | H=1.97 | 0.373 | 0.918 |  |
|  | Secondary Hospital (n=27) | 3.42±0.86 |  |  |  |  |
|  | Teritary HospitalA (n=94) | 3.33 (2.75-4) |  |  |  |  |
| Needs_Clinical_Decision | Primary Hospital (n=13) | 4.33 (3.67-5) | H=1.5 | 0.473 | 0.918 |  |
|  | Secondary Hospital (n=27) | 4 (3.67-4.67) |  |  |  |  |
|  | Teritary HospitalA (n=94) | 4 (3.33-4.67) |  |  |  |  |
| Needs_Routine_Tasks | Primary Hospital (n=13) | 3.6±1.13 | H=0.12 | 0.941 | 0.965 |  |
|  | Secondary Hospital (n=27) | 3.75 (3.25-4.12) |  |  |  |  |
|  | Teritary HospitalA (n=94) | 3.75 (3-4.25) |  |  |  |  |
| Needs_Interaction | Primary Hospital (n=13) | 3.58±1.37 | H=1.6 | 0.45 | 0.918 |  |
|  | Secondary Hospital (n=27) | 3.15±1.06 |  |  |  |  |
|  | Teritary HospitalA (n=94) | 3.25 (2.5-4) |  |  |  |  |
| Medical Needs | Primary Hospital (n=13) | 3.78±0.95 | H=0.4 | 0.817 | 0.964 |  |
|  | Secondary Hospital (n=27) | 3.6±0.67 |  |  |  |  |
|  | Teritary HospitalA (n=94) | 3.78 (3-4.22) |  |  |  |  |
| Potential_Clinical_Efficiency | Primary Hospital (n=13) | 4.33 (4-5) | H=0.68 | 0.712 | 0.918 |  |
|  | Secondary Hospital (n=27) | 4 (3.67-4.83) |  |  |  |  |
|  | Teritary HospitalA (n=94) | 4 (3.38-4.83) |  |  |  |  |
| Potential_Humanistic_Care | Primary Hospital (n=13) | 3.69±1.17 | H=0.91 | 0.634 | 0.918 |  |
|  | Secondary Hospital (n=27) | 3.48±1.22 |  |  |  |  |
|  | Teritary HospitalA (n=94) | 3.67 (2.67-4.33) |  |  |  |  |
| Medical Potential | Primary Hospital (n=13) | 3.96±1.02 | H=1.23 | 0.54 | 0.918 |  |
|  | Secondary Hospital (n=27) | 3.94±0.75 |  |  |  |  |
|  | Teritary HospitalA (n=94) | 3.78 (3.25-4.56) |  |  |  |  |
| Optimization_Clinical_Efficiency | Primary Hospital (n=13) | 4.33 (4-5) | H=0.72 | 0.698 | 0.918 |  |
|  | Secondary Hospital (n=27) | 4 (3.5-4.92) |  |  |  |  |
|  | Teritary HospitalA (n=94) | 4.17 (3.67-5) |  |  |  |  |
| Optimization_Humanistic_Care | Primary Hospital (n=13) | 3.62±1.33 | H=0.85 | 0.652 | 0.918 |  |
|  | Secondary Hospital (n=27) | 3.44±1.17 |  |  |  |  |
|  | Teritary HospitalA (n=94) | 3.83 (3-5) |  |  |  |  |
| Medical Optimization | Primary Hospital (n=13) | 4.11 (3.44-5) | H=0.65 | 0.722 | 0.918 |  |
|  | Secondary Hospital (n=27) | 3.88±0.82 |  |  |  |  |
|  | Teritary HospitalA (n=94) | 4 (3.39-4.89) |  |  |  |  |
| Journals | Primary Hospital (n=13) | 0 (0-0) | H=3.87 | 0.145 | 0.918 |  |
|  | Secondary Hospital (n=27) | 0 (0-1) |  |  |  |  |
|  | Teritary HospitalA (n=94) | 0 (0-1) |  |  |  |  |
| Online Courses | Primary Hospital (n=13) | 0 (0-0) | H=5.37 | 0.068 | 0.918 |  |
|  | Secondary Hospital (n=27) | 0 (0-1) |  |  |  |  |
|  | Teritary HospitalA (n=94) | 0 (0-1) |  |  |  |  |
| Hospital Training | Primary Hospital (n=13) | 0 (0-0) | H=0.75 | 0.687 | 0.918 |  |
|  | Secondary Hospital (n=27) | 0 (0-0) |  |  |  |  |
|  | Teritary HospitalA (n=94) | 0 (0-1) |  |  |  |  |
| Social Media | Primary Hospital (n=13) | 1 (1-1) | H=0.11 | 0.948 | 0.965 |  |
|  | Secondary Hospital (n=27) | 1 (1-1) |  |  |  |  |
|  | Teritary HospitalA (n=94) | 1 (1-1) |  |  |  |  |
| Conferences | Primary Hospital (n=13) | 0 (0-0) | H=0.8 | 0.669 | 0.918 |  |
|  | Secondary Hospital (n=27) | 0 (0-0) |  |  |  |  |
|  | Teritary HospitalA (n=94) | 0 (0-0) |  |  |  |  |
| Colleague Discussions | Primary Hospital (n=13) | 1 (0-1) | H=0.71 | 0.7 | 0.918 |  |
|  | Secondary Hospital (n=27) | 1 (0-1) |  |  |  |  |
|  | Teritary HospitalA (n=94) | 1 (0-1) |  |  |  |  |
| News & Websites | Primary Hospital (n=13) | 1 (0-1) | H=1.38 | 0.501 | 0.918 |  |
|  | Secondary Hospital (n=27) | 1 (0-1) |  |  |  |  |
|  | Teritary HospitalA (n=94) | 1 (0-1) |  |  |  |  |
| Guidelines | Primary Hospital (n=13) | 0 (0-0) | H=1.8 | 0.407 | 0.918 |  |
|  | Secondary Hospital (n=27) | 0 (0-0) |  |  |  |  |
|  | Teritary HospitalA (n=94) | 0 (0-0) |  |  |  |  |
| Books | Primary Hospital (n=13) | 0 (0-0) | H=2.01 | 0.367 | 0.918 |  |
|  | Secondary Hospital (n=27) | 0 (0-0) |  |  |  |  |
|  | Teritary HospitalA (n=94) | 0 (0-0) |  |  |  |  |
| Vendor Docs | Primary Hospital (n=13) | 0 (0-0) | H=1.88 | 0.39 | 0.918 |  |
|  | Secondary Hospital (n=27) | 0 (0-0) |  |  |  |  |
|  | Teritary HospitalA (n=94) | 0 (0-0) |  |  |  |  |
| Other | Primary Hospital (n=13) | 0 (0-0) | H=0.56 | 0.754 | 0.918 |  |
|  | Secondary Hospital (n=27) | 0 (0-0) |  |  |  |  |
|  | Teritary HospitalA (n=94) | 0 (0-0) |  |  |  |  |
| Understanding | Primary Hospital (n=13) | 3 (2-3) | H=2.25 | 0.324 | 0.918 |  |
|  | Secondary Hospital (n=27) | 3 (2-4) |  |  |  |  |
|  | Teritary HospitalA (n=94) | 3 (2-3) |  |  |  |  |
| Reading Articles | Primary Hospital (n=13) | 1 (1-2) | H=8.09 | 0.018 | 0.918 | Teritary HospitalA vs Primary Hospital |
|  | Secondary Hospital (n=27) | 2 (2-3) |  |  |  |  |
|  | Teritary HospitalA (n=94) | 3 (2-4) |  |  |  |  |
| Effective Use | Primary Hospital (n=13) | 2.77±1.17 | H=2.02 | 0.364 | 0.918 |  |
|  | Secondary Hospital (n=27) | 3 (2-4.5) |  |  |  |  |
|  | Teritary HospitalA (n=94) | 3 (2-4) |  |  |  |  |
| Access & Use | Primary Hospital (n=13) | 2.62±1.12 | H=4.54 | 0.104 | 0.918 |  |
|  | Secondary Hospital (n=27) | 3 (2-4) |  |  |  |  |
|  | Teritary HospitalA (n=94) | 3 (2-4) |  |  |  |  |
| Active Seeking | Primary Hospital (n=13) | 3±1.15 | H=1.79 | 0.409 | 0.918 |  |
|  | Secondary Hospital (n=27) | 4 (2.5-4) |  |  |  |  |
|  | Teritary HospitalA (n=94) | 3 (3-4) |  |  |  |  |
| Future Importance | Primary Hospital (n=13) | 4 (3-4) | H=1.25 | 0.535 | 0.918 |  |
|  | Secondary Hospital (n=27) | 4 (3-5) |  |  |  |  |
|  | Teritary HospitalA (n=94) | 4 (3-5) |  |  |  |  |
| Needs_Patient Management | Primary Hospital (n=13) | 4 (4-5) | H=1.07 | 0.585 | 0.918 |  |
|  | Secondary Hospital (n=27) | 4 (3-5) |  |  |  |  |
|  | Teritary HospitalA (n=94) | 4 (3-5) |  |  |  |  |
| Needs_Medical History Collection | Primary Hospital (n=13) | 3.54±1.33 | H=0.26 | 0.878 | 0.964 |  |
|  | Secondary Hospital (n=27) | 4 (3-4) |  |  |  |  |
|  | Teritary HospitalA (n=94) | 3 (3-4) |  |  |  |  |
| Needs_Mental Status Examination | Primary Hospital (n=13) | 2 (1-4) | H=1.2 | 0.55 | 0.918 |  |
|  | Secondary Hospital (n=27) | 3 (2-3.5) |  |  |  |  |
|  | Teritary HospitalA (n=94) | 3 (2-4) |  |  |  |  |
| Needs_Medical Documentation Writing | Primary Hospital (n=13) | 4 (4-5) | H=0.18 | 0.916 | 0.965 |  |
|  | Secondary Hospital (n=27) | 4 (4-5) |  |  |  |  |
|  | Teritary HospitalA (n=94) | 4 (4-5) |  |  |  |  |
| Needs_Diagnostic Assistance | Primary Hospital (n=13) | 5 (3-5) | H=1.28 | 0.528 | 0.918 |  |
|  | Secondary Hospital (n=27) | 4 (4-5) |  |  |  |  |
|  | Teritary HospitalA (n=94) | 4 (3-5) |  |  |  |  |
| Needs_Treatment Planning and Outcome Prediction | Primary Hospital (n=13) | 4 (4-5) | H=0.91 | 0.633 | 0.918 |  |
|  | Secondary Hospital (n=27) | 4 (3.5-5) |  |  |  |  |
|  | Teritary HospitalA (n=94) | 4 (3-5) |  |  |  |  |
| Needs_Risk Assessment and Prognosis Estimation | Primary Hospital (n=13) | 4 (4-5) | H=1.32 | 0.516 | 0.918 |  |
|  | Secondary Hospital (n=27) | 4 (3.5-4.5) |  |  |  |  |
|  | Teritary HospitalA (n=94) | 4 (3-5) |  |  |  |  |
| Needs_Doctor-Patient Communication | Primary Hospital (n=13) | 3.62±1.33 | H=1.84 | 0.399 | 0.918 |  |
|  | Secondary Hospital (n=27) | 3 (2.5-4) |  |  |  |  |
|  | Teritary HospitalA (n=94) | 3 (2-4) |  |  |  |  |
| Needs_Psychological Interventions | Primary Hospital (n=13) | 4 (3-5) | H=0.92 | 0.63 | 0.918 |  |
|  | Secondary Hospital (n=27) | 3 (3-4) |  |  |  |  |
|  | Teritary HospitalA (n=94) | 3 (2-4) |  |  |  |  |
| Potential_Patient Management | Primary Hospital (n=13) | 4 (4-5) | H=2.25 | 0.325 | 0.918 |  |
|  | Secondary Hospital (n=27) | 4 (3-4) |  |  |  |  |
|  | Teritary HospitalA (n=94) | 4 (3-5) |  |  |  |  |
| Potential_Medical History Collection | Primary Hospital (n=13) | 4 (3-5) | H=1.36 | 0.507 | 0.918 |  |
|  | Secondary Hospital (n=27) | 4 (3-5) |  |  |  |  |
|  | Teritary HospitalA (n=94) | 4 (3-5) |  |  |  |  |
| Potential_Mental Status Examination | Primary Hospital (n=13) | 4 (2-5) | H=0.15 | 0.928 | 0.965 |  |
|  | Secondary Hospital (n=27) | 3 (2.5-5) |  |  |  |  |
|  | Teritary HospitalA (n=94) | 3 (3-4) |  |  |  |  |
| Potential_Medical Documentation Writing | Primary Hospital (n=13) | 5 (4-5) | H=0.3 | 0.862 | 0.964 |  |
|  | Secondary Hospital (n=27) | 5 (4-5) |  |  |  |  |
|  | Teritary HospitalA (n=94) | 5 (4-5) |  |  |  |  |
| Potential_Diagnostic Assistance | Primary Hospital (n=13) | 4 (3-5) | H=0.91 | 0.634 | 0.918 |  |
|  | Secondary Hospital (n=27) | 4 (4-5) |  |  |  |  |
|  | Teritary HospitalA (n=94) | 4 (3-5) |  |  |  |  |
| Potential_Treatment Planning and Outcome Prediction | Primary Hospital (n=13) | 5 (4-5) | H=1.88 | 0.39 | 0.918 |  |
|  | Secondary Hospital (n=27) | 4 (4-5) |  |  |  |  |
|  | Teritary HospitalA (n=94) | 4 (3-5) |  |  |  |  |
| Potential_Risk Assessment and Prognosis Estimation | Primary Hospital (n=13) | 5 (4-5) | H=2.18 | 0.336 | 0.918 |  |
|  | Secondary Hospital (n=27) | 4 (4-5) |  |  |  |  |
|  | Teritary HospitalA (n=94) | 4 (3.25-5) |  |  |  |  |
| Potential_Doctor-Patient Communication | Primary Hospital (n=13) | 4 (3-5) | H=1.48 | 0.477 | 0.918 |  |
|  | Secondary Hospital (n=27) | 3 (2.5-5) |  |  |  |  |
|  | Teritary HospitalA (n=94) | 4 (2-4.75) |  |  |  |  |
| Potential_Psychological Interventions | Primary Hospital (n=13) | 4 (3-5) | H=1.82 | 0.402 | 0.918 |  |
|  | Secondary Hospital (n=27) | 4 (3-5) |  |  |  |  |
|  | Teritary HospitalA (n=94) | 4 (3-4) |  |  |  |  |
| Optimization_Patient Management | Primary Hospital (n=13) | 5 (4-5) | H=1.04 | 0.595 | 0.918 |  |
|  | Secondary Hospital (n=27) | 4 (4-5) |  |  |  |  |
|  | Teritary HospitalA (n=94) | 4 (3.25-5) |  |  |  |  |
| Optimization_Medical History Collection | Primary Hospital (n=13) | 4 (3-5) | H=0.05 | 0.976 | 0.976 |  |
|  | Secondary Hospital (n=27) | 4 (3.5-5) |  |  |  |  |
|  | Teritary HospitalA (n=94) | 4 (3-5) |  |  |  |  |
| Optimization_Mental Status Examination | Primary Hospital (n=13) | 4 (2-5) | H=1.77 | 0.413 | 0.918 |  |
|  | Secondary Hospital (n=27) | 3 (2-4) |  |  |  |  |
|  | Teritary HospitalA (n=94) | 4 (3-5) |  |  |  |  |
| Optimization_Medical Documentation Writing | Primary Hospital (n=13) | 4 (4-5) | H=0.6 | 0.741 | 0.918 |  |
|  | Secondary Hospital (n=27) | 5 (4-5) |  |  |  |  |
|  | Teritary HospitalA (n=94) | 5 (4-5) |  |  |  |  |
| Optimization_Diagnostic Assistance | Primary Hospital (n=13) | 5 (3-5) | H=0.34 | 0.844 | 0.964 |  |
|  | Secondary Hospital (n=27) | 4 (4-5) |  |  |  |  |
|  | Teritary HospitalA (n=94) | 4 (3-5) |  |  |  |  |
| Optimization_Treatment Planning and Outcome Prediction | Primary Hospital (n=13) | 5 (4-5) | H=1.21 | 0.547 | 0.918 |  |
|  | Secondary Hospital (n=27) | 4 (4-5) |  |  |  |  |
|  | Teritary HospitalA (n=94) | 4 (3-5) |  |  |  |  |
| Optimization_Risk Assessment and Prognosis Estimation | Primary Hospital (n=13) | 5 (4-5) | H=1.08 | 0.583 | 0.918 |  |
|  | Secondary Hospital (n=27) | 4 (3.5-5) |  |  |  |  |
|  | Teritary HospitalA (n=94) | 4 (4-5) |  |  |  |  |
| Optimization_Doctor-Patient Communication | Primary Hospital (n=13) | 4 (4-5) | H=2.24 | 0.326 | 0.918 |  |
|  | Secondary Hospital (n=27) | 4 (2-4) |  |  |  |  |
|  | Teritary HospitalA (n=94) | 4 (3-5) |  |  |  |  |
| Optimization_Psychological Interventions | Primary Hospital (n=13) | 4 (3-5) | H=0.34 | 0.843 | 0.964 |  |
|  | Secondary Hospital (n=27) | 4 (3-4.5) |  |  |  |  |
|  | Teritary HospitalA (n=94) | 4 (3-5) |  |  |  |  |

Table S5：Group differences among titles

| **Item** | **Group** | **Descriptive Statistics** | **Statistic** | **Raw P-value** | **P-value (FDR)** | **Post-hoc Test** |
| --- | --- | --- | --- | --- | --- | --- |
| AI Exposure & Learning | Junior (n=42) | 3.1±1.12 | F=0.7 | 0.554 | 0.713 |  |
|  | Intermediate (n=38) | 2.8±1.04 |  |  |  |  |
|  | Associate Senior (n=27) | 3.12±1.08 |  |  |  |  |
|  | Senior (n=12) | 3.11±1.1 |  |  |  |  |
| AI Trust & Expectations | Junior (n=42) | 3.37±0.92 | F=0.42 | 0.741 | 0.814 |  |
|  | Intermediate (n=38) | 3.2±0.88 |  |  |  |  |
|  | Associate Senior (n=27) | 3.42±0.84 |  |  |  |  |
|  | Senior (n=12) | 3.39±0.8 |  |  |  |  |
| Needs_Clinical_Decision | Junior (n=42) | 4 (3-5) | H=5.19 | 0.158 | 0.504 |  |
|  | Intermediate (n=38) | 4 (3.17-4.67) |  |  |  |  |
|  | Associate Senior (n=27) | 3.96±0.68 |  |  |  |  |
|  | Senior (n=12) | 5 (4-5) |  |  |  |  |
| Needs_Routine_Tasks | Junior (n=42) | 3.51±1.05 | H=2.84 | 0.417 | 0.677 |  |
|  | Intermediate (n=38) | 3.75 (3-4.19) |  |  |  |  |
|  | Associate Senior (n=27) | 3.57±0.73 |  |  |  |  |
|  | Senior (n=12) | 3.96±0.98 |  |  |  |  |
| Needs_Interaction | Junior (n=42) | 3 (2-4) | H=4.18 | 0.243 | 0.542 |  |
|  | Intermediate (n=38) | 3 (2.5-4) |  |  |  |  |
|  | Associate Senior (n=27) | 4 (3.5-4.25) |  |  |  |  |
|  | Senior (n=12) | 3.29±1.41 |  |  |  |  |
| Medical Needs | Junior (n=42) | 3.54±0.97 | F=1.25 | 0.295 | 0.551 |  |
|  | Intermediate (n=38) | 3.48±0.87 |  |  |  |  |
|  | Associate Senior (n=27) | 3.72±0.63 |  |  |  |  |
|  | Senior (n=12) | 3.97±0.84 |  |  |  |  |
| Potential_Clinical_Efficiency | Junior (n=42) | 4.08 (3.33-4.83) | H=5.16 | 0.16 | 0.504 |  |
|  | Intermediate (n=38) | 4 (3.67-4.67) |  |  |  |  |
|  | Associate Senior (n=27) | 4 (3.58-4.83) |  |  |  |  |
|  | Senior (n=12) | 4.83 (4.46-5) |  |  |  |  |
| Potential_Humanistic_Care | Junior (n=42) | 3.67 (2.67-4.25) | H=1.49 | 0.684 | 0.814 |  |
|  | Intermediate (n=38) | 3.39±1.11 |  |  |  |  |
|  | Associate Senior (n=27) | 3.67 (2.67-4.33) |  |  |  |  |
|  | Senior (n=12) | 3.72±1.31 |  |  |  |  |
| Medical Potential | Junior (n=42) | 3.76±0.94 | F=1.43 | 0.238 | 0.542 |  |
|  | Intermediate (n=38) | 3.78±0.9 |  |  |  |  |
|  | Associate Senior (n=27) | 3.97±0.77 |  |  |  |  |
|  | Senior (n=12) | 4.3±0.75 |  |  |  |  |
| Optimization_Clinical_Efficiency | Junior (n=42) | 4 (3.5-4.96) | H=5.57 | 0.135 | 0.504 |  |
|  | Intermediate (n=38) | 4.17 (3.88-5) |  |  |  |  |
|  | Associate Senior (n=27) | 4.33 (3.58-5) |  |  |  |  |
|  | Senior (n=12) | 5 (4.54-5) |  |  |  |  |
| Optimization_Humanistic_Care | Junior (n=42) | 3.39±1.16 | H=3.74 | 0.291 | 0.551 |  |
|  | Intermediate (n=38) | 3.67 (2.42-5) |  |  |  |  |
|  | Associate Senior (n=27) | 4 (3.67-4.83) |  |  |  |  |
|  | Senior (n=12) | 4.67 (2.75-5) |  |  |  |  |
| Medical Optimization | Junior (n=42) | 3.81±0.87 | H=4.19 | 0.242 | 0.542 |  |
|  | Intermediate (n=38) | 4 (3.39-4.89) |  |  |  |  |
|  | Associate Senior (n=27) | 4.22 (3.72-4.94) |  |  |  |  |
|  | Senior (n=12) | 4.78 (3.47-5) |  |  |  |  |
| Journals | Junior (n=42) | 0 (0-1) | H=3.74 | 0.291 | 0.551 |  |
|  | Intermediate (n=38) | 0 (0-0.75) |  |  |  |  |
|  | Associate Senior (n=27) | 0 (0-1) |  |  |  |  |
|  | Senior (n=12) | 0 (0-0.25) |  |  |  |  |
| Online Courses | Junior (n=42) | 0 (0-1) | H=3.71 | 0.295 | 0.551 |  |
|  | Intermediate (n=38) | 0 (0-0) |  |  |  |  |
|  | Associate Senior (n=27) | 0 (0-1) |  |  |  |  |
|  | Senior (n=12) | 0.5 (0-1) |  |  |  |  |
| Hospital Training | Junior (n=42) | 0 (0-1) | H=5.57 | 0.134 | 0.504 |  |
|  | Intermediate (n=38) | 0 (0-0) |  |  |  |  |
|  | Associate Senior (n=27) | 0 (0-1) |  |  |  |  |
|  | Senior (n=12) | 0 (0-0.25) |  |  |  |  |
| Social Media | Junior (n=42) | 1 (0-1) | H=4.51 | 0.211 | 0.542 |  |
|  | Intermediate (n=38) | 1 (1-1) |  |  |  |  |
|  | Associate Senior (n=27) | 1 (1-1) |  |  |  |  |
|  | Senior (n=12) | 1 (1-1) |  |  |  |  |
| Conferences | Junior (n=42) | 0 (0-0) | H=1.36 | 0.714 | 0.814 |  |
|  | Intermediate (n=38) | 0 (0-0) |  |  |  |  |
|  | Associate Senior (n=27) | 0 (0-0) |  |  |  |  |
|  | Senior (n=12) | 0 (0-0) |  |  |  |  |
| Colleague Discussions | Junior (n=42) | 1 (0-1) | H=2.87 | 0.412 | 0.677 |  |
|  | Intermediate (n=38) | 1 (0-1) |  |  |  |  |
|  | Associate Senior (n=27) | 1 (0.5-1) |  |  |  |  |
|  | Senior (n=12) | 1 (0-1) |  |  |  |  |
| News & Websites | Junior (n=42) | 0 (0-1) | H=6.24 | 0.101 | 0.504 |  |
|  | Intermediate (n=38) | 1 (0-1) |  |  |  |  |
|  | Associate Senior (n=27) | 1 (0-1) |  |  |  |  |
|  | Senior (n=12) | 1 (1-1) |  |  |  |  |
| Guidelines | Junior (n=42) | 0 (0-0) | H=7.1 | 0.069 | 0.504 |  |
|  | Intermediate (n=38) | 0 (0-0) |  |  |  |  |
|  | Associate Senior (n=27) | 0 (0-0) |  |  |  |  |
|  | Senior (n=12) | 0 (0-1) |  |  |  |  |
| Books | Junior (n=42) | 0 (0-0) | H=1.85 | 0.604 | 0.736 |  |
|  | Intermediate (n=38) | 0 (0-0) |  |  |  |  |
|  | Associate Senior (n=27) | 0 (0-0) |  |  |  |  |
|  | Senior (n=12) | 0 (0-0) |  |  |  |  |
| Vendor Docs | Junior (n=42) | 0 (0-0) | H=0.22 | 0.975 | 0.975 |  |
|  | Intermediate (n=38) | 0 (0-0) |  |  |  |  |
|  | Associate Senior (n=27) | 0 (0-0) |  |  |  |  |
|  | Senior (n=12) | 0 (0-0) |  |  |  |  |
| Other | Junior (n=42) | 0 (0-0) | H=2.77 | 0.429 | 0.677 |  |
|  | Intermediate (n=38) | 0 (0-0) |  |  |  |  |
|  | Associate Senior (n=27) | 0 (0-0) |  |  |  |  |
|  | Senior (n=12) | 0 (0-0) |  |  |  |  |
| Understanding | Junior (n=42) | 3 (2-4) | H=2.13 | 0.547 | 0.713 |  |
|  | Intermediate (n=38) | 3 (2-3) |  |  |  |  |
|  | Associate Senior (n=27) | 3 (2-3.5) |  |  |  |  |
|  | Senior (n=12) | 2.75±1.06 |  |  |  |  |
| Reading Articles | Junior (n=42) | 2 (1.25-3) | H=1.16 | 0.762 | 0.814 |  |
|  | Intermediate (n=38) | 2 (1-3) |  |  |  |  |
|  | Associate Senior (n=27) | 2 (2-3.5) |  |  |  |  |
|  | Senior (n=12) | 2.75±1.29 |  |  |  |  |
| Effective Use | Junior (n=42) | 3 (3-4) | H=2.06 | 0.56 | 0.713 |  |
|  | Intermediate (n=38) | 3 (2-4) |  |  |  |  |
|  | Associate Senior (n=27) | 3 (2-4) |  |  |  |  |
|  | Senior (n=12) | 2.92±1.16 |  |  |  |  |
| Access & Use | Junior (n=42) | 3 (3-4.75) | H=3.39 | 0.335 | 0.605 |  |
|  | Intermediate (n=38) | 2.5 (2-4) |  |  |  |  |
|  | Associate Senior (n=27) | 3 (2-4) |  |  |  |  |
|  | Senior (n=12) | 3.17±1.4 |  |  |  |  |
| Active Seeking | Junior (n=42) | 3 (3-4) | H=1.91 | 0.591 | 0.736 |  |
|  | Intermediate (n=38) | 3 (2-4) |  |  |  |  |
|  | Associate Senior (n=27) | 4 (3-4) |  |  |  |  |
|  | Senior (n=12) | 3 (3-4) |  |  |  |  |
| Future Importance | Junior (n=42) | 4 (3-5) | H=10.92 | 0.012 | 0.404 | 无显著两两差异 |
|  | Intermediate (n=38) | 4 (3-4.75) |  |  |  |  |
|  | Associate Senior (n=27) | 5 (4-5) |  |  |  |  |
|  | Senior (n=12) | 5 (4-5) |  |  |  |  |
| Needs_Patient Management | Junior (n=42) | 4 (3-5) | H=5.75 | 0.124 | 0.504 |  |
|  | Intermediate (n=38) | 4 (3-4) |  |  |  |  |
|  | Associate Senior (n=27) | 4 (3.5-5) |  |  |  |  |
|  | Senior (n=12) | 5 (4-5) |  |  |  |  |
| Needs_Medical History Collection | Junior (n=42) | 3 (3-4) | H=2.3 | 0.513 | 0.713 |  |
|  | Intermediate (n=38) | 4 (2.25-4) |  |  |  |  |
|  | Associate Senior (n=27) | 3 (3-4) |  |  |  |  |
|  | Senior (n=12) | 3.83±1.11 |  |  |  |  |
| Needs_Mental Status Examination | Junior (n=42) | 3 (2-4) | H=0.85 | 0.837 | 0.868 |  |
|  | Intermediate (n=38) | 3 (2-4) |  |  |  |  |
|  | Associate Senior (n=27) | 3 (2-3) |  |  |  |  |
|  | Senior (n=12) | 3.17±1.64 |  |  |  |  |
| Needs_Medical Documentation Writing | Junior (n=42) | 4 (4-5) | H=6.06 | 0.109 | 0.504 |  |
|  | Intermediate (n=38) | 4 (3-5) |  |  |  |  |
|  | Associate Senior (n=27) | 4 (4-5) |  |  |  |  |
|  | Senior (n=12) | 5 (4-5) |  |  |  |  |
| Needs_Diagnostic Assistance | Junior (n=42) | 4 (3-5) | H=5.14 | 0.162 | 0.504 |  |
|  | Intermediate (n=38) | 4 (3.25-4.75) |  |  |  |  |
|  | Associate Senior (n=27) | 4 (3.5-5) |  |  |  |  |
|  | Senior (n=12) | 5 (4-5) |  |  |  |  |
| Needs_Treatment Planning and Outcome Prediction | Junior (n=42) | 4 (3-5) | H=2.66 | 0.448 | 0.677 |  |
|  | Intermediate (n=38) | 4 (4-5) |  |  |  |  |
|  | Associate Senior (n=27) | 4 (4-4) |  |  |  |  |
|  | Senior (n=12) | 5 (4-5) |  |  |  |  |
| Needs_Risk Assessment and Prognosis Estimation | Junior (n=42) | 4 (3-5) | H=4.09 | 0.252 | 0.542 |  |
|  | Intermediate (n=38) | 4 (3-4.75) |  |  |  |  |
|  | Associate Senior (n=27) | 4 (4-4) |  |  |  |  |
|  | Senior (n=12) | 5 (4-5) |  |  |  |  |
| Needs_Doctor-Patient Communication | Junior (n=42) | 3 (2-4) | H=4.98 | 0.173 | 0.51 |  |
|  | Intermediate (n=38) | 3 (2-4) |  |  |  |  |
|  | Associate Senior (n=27) | 4 (3-4) |  |  |  |  |
|  | Senior (n=12) | 3.25±1.48 |  |  |  |  |
| Needs_Psychological Interventions | Junior (n=42) | 3 (2-4) | H=2.68 | 0.443 | 0.677 |  |
|  | Intermediate (n=38) | 3 (2-4) |  |  |  |  |
|  | Associate Senior (n=27) | 4 (3-4) |  |  |  |  |
|  | Senior (n=12) | 3.33±1.44 |  |  |  |  |
| Potential_Patient Management | Junior (n=42) | 4 (3-5) | H=6.35 | 0.096 | 0.504 |  |
|  | Intermediate (n=38) | 4 (3.25-5) |  |  |  |  |
|  | Associate Senior (n=27) | 4 (4-5) |  |  |  |  |
|  | Senior (n=12) | 5 (4.75-5) |  |  |  |  |
| Potential_Medical History Collection | Junior (n=42) | 4 (3-5) | H=2.15 | 0.541 | 0.713 |  |
|  | Intermediate (n=38) | 4 (3-5) |  |  |  |  |
|  | Associate Senior (n=27) | 4 (3-4) |  |  |  |  |
|  | Senior (n=12) | 5 (3.5-5) |  |  |  |  |
| Potential_Mental Status Examination | Junior (n=42) | 3.5 (3-5) | H=0.46 | 0.928 | 0.945 |  |
|  | Intermediate (n=38) | 3 (2-4) |  |  |  |  |
|  | Associate Senior (n=27) | 3 (3-4) |  |  |  |  |
|  | Senior (n=12) | 4 (2.5-5) |  |  |  |  |
| Potential_Medical Documentation Writing | Junior (n=42) | 5 (4-5) | H=6.05 | 0.109 | 0.504 |  |
|  | Intermediate (n=38) | 4 (3-5) |  |  |  |  |
|  | Associate Senior (n=27) | 5 (4-5) |  |  |  |  |
|  | Senior (n=12) | 5 (5-5) |  |  |  |  |
| Potential_Diagnostic Assistance | Junior (n=42) | 4 (3-5) | H=10.12 | 0.018 | 0.404 | Senior vs Junior |
|  | Intermediate (n=38) | 4 (4-5) |  |  |  |  |
|  | Associate Senior (n=27) | 5 (4-5) |  |  |  |  |
|  | Senior (n=12) | 5 (4.75-5) |  |  |  |  |
| Potential_Treatment Planning and Outcome Prediction | Junior (n=42) | 4 (3-5) | H=2.38 | 0.498 | 0.713 |  |
|  | Intermediate (n=38) | 4 (4-5) |  |  |  |  |
|  | Associate Senior (n=27) | 4 (3-5) |  |  |  |  |
|  | Senior (n=12) | 5 (4-5) |  |  |  |  |
| Potential_Risk Assessment and Prognosis Estimation | Junior (n=42) | 4 (4-5) | H=4.31 | 0.229 | 0.542 |  |
|  | Intermediate (n=38) | 4 (4-5) |  |  |  |  |
|  | Associate Senior (n=27) | 4 (4-5) |  |  |  |  |
|  | Senior (n=12) | 5 (4-5) |  |  |  |  |
| Potential_Doctor-Patient Communication | Junior (n=42) | 3 (2.25-4) | H=5.21 | 0.157 | 0.504 |  |
|  | Intermediate (n=38) | 3.5 (2-4.75) |  |  |  |  |
|  | Associate Senior (n=27) | 4 (3-5) |  |  |  |  |
|  | Senior (n=12) | 5 (3.75-5) |  |  |  |  |
| Potential_Psychological Interventions | Junior (n=42) | 3 (3-4.75) | H=1.16 | 0.763 | 0.814 |  |
|  | Intermediate (n=38) | 4 (3-4.75) |  |  |  |  |
|  | Associate Senior (n=27) | 4 (3-4) |  |  |  |  |
|  | Senior (n=12) | 4 (2.75-5) |  |  |  |  |
| Optimization_Patient Management | Junior (n=42) | 4 (4-5) | H=2.5 | 0.476 | 0.701 |  |
|  | Intermediate (n=38) | 4.5 (4-5) |  |  |  |  |
|  | Associate Senior (n=27) | 4 (3-5) |  |  |  |  |
|  | Senior (n=12) | 5 (4-5) |  |  |  |  |
| Optimization_Medical History Collection | Junior (n=42) | 4 (3-5) | H=1.13 | 0.77 | 0.814 |  |
|  | Intermediate (n=38) | 4 (3-5) |  |  |  |  |
|  | Associate Senior (n=27) | 4 (3-5) |  |  |  |  |
|  | Senior (n=12) | 5 (3.75-5) |  |  |  |  |
| Optimization_Mental Status Examination | Junior (n=42) | 4 (3-5) | H=1.17 | 0.759 | 0.814 |  |
|  | Intermediate (n=38) | 4 (2-5) |  |  |  |  |
|  | Associate Senior (n=27) | 4 (3-5) |  |  |  |  |
|  | Senior (n=12) | 4.5 (2.75-5) |  |  |  |  |
| Optimization_Medical Documentation Writing | Junior (n=42) | 4 (4-5) | H=4.22 | 0.239 | 0.542 |  |
|  | Intermediate (n=38) | 5 (4-5) |  |  |  |  |
|  | Associate Senior (n=27) | 5 (4-5) |  |  |  |  |
|  | Senior (n=12) | 5 (5-5) |  |  |  |  |
| Optimization_Diagnostic Assistance | Junior (n=42) | 4 (3-5) | H=8.67 | 0.034 | 0.477 | Senior vs Junior |
|  | Intermediate (n=38) | 4 (4-5) |  |  |  |  |
|  | Associate Senior (n=27) | 5 (4-5) |  |  |  |  |
|  | Senior (n=12) | 5 (5-5) |  |  |  |  |
| Optimization_Treatment Planning and Outcome Prediction | Junior (n=42) | 4 (3-5) | H=9.66 | 0.022 | 0.404 | Senior vs Junior |
|  | Intermediate (n=38) | 4 (4-5) |  |  |  |  |
|  | Associate Senior (n=27) | 4 (4-5) |  |  |  |  |
|  | Senior (n=12) | 5 (5-5) |  |  |  |  |
| Optimization_Risk Assessment and Prognosis Estimation | Junior (n=42) | 4 (3-5) | H=7.86 | 0.049 | 0.504 | 无显著两两差异 |
|  | Intermediate (n=38) | 4 (4-5) |  |  |  |  |
|  | Associate Senior (n=27) | 5 (4-5) |  |  |  |  |
|  | Senior (n=12) | 5 (5-5) |  |  |  |  |
| Optimization_Doctor-Patient Communication | Junior (n=42) | 3.5 (3-4) | H=3.21 | 0.36 | 0.63 |  |
|  | Intermediate (n=38) | 4 (2-5) |  |  |  |  |
|  | Associate Senior (n=27) | 4 (4-5) |  |  |  |  |
|  | Senior (n=12) | 5 (2-5) |  |  |  |  |
| Optimization_Psychological Interventions | Junior (n=42) | 3 (3-4) | H=5.48 | 0.14 | 0.504 |  |
|  | Intermediate (n=38) | 4 (2.25-5) |  |  |  |  |
|  | Associate Senior (n=27) | 4 (4-5) |  |  |  |  |
|  | Senior (n=12) | 4.5 (2.75-5) |  |  |  |  |

Table S6：Group differences between whether AI training

| **Item** | **Group** | **Descriptive Statistics** | **Statistic** | **Raw P-value** | **P-value (FDR)** | **Post-hoc Test** |
| --- | --- | --- | --- | --- | --- | --- |
| AI Exposure & Learning | No (n=119) | 2.67 (2.33-3.67) | Z=366.5 | < 0.001 | 0.005 |  |
|  | Yes (n=15) | 4.33 (4-4.83) |  |  |  |  |
| AI Trust & Expectations | No (n=119) | 3.33 (2.67-3.67) | Z=454 | 0.002 | 0.021 |  |
|  | Yes (n=15) | 3.98±0.85 |  |  |  |  |
| Needs_Clinical_Decision | No (n=119) | 4 (3.33-4.83) | Z=943 | 0.721 | 0.9 |  |
|  | Yes (n=15) | 3.93±0.81 |  |  |  |  |
| Needs_Routine_Tasks | No (n=119) | 3.5 (3-4.12) | Z=666.5 | 0.11 | 0.474 |  |
|  | Yes (n=15) | 4.25 (3.62-4.25) |  |  |  |  |
| Needs_Interaction | No (n=119) | 3.5 (2.5-4) | Z=962.5 | 0.621 | 0.9 |  |
|  | Yes (n=15) | 3.1±1.09 |  |  |  |  |
| Medical Needs | No (n=119) | 3.6±0.85 | Z=838.5 | 0.705 | 0.9 |  |
|  | Yes (n=15) | 3.78 (3.67-4.06) |  |  |  |  |
| Potential_Clinical_Efficiency | No (n=119) | 4 (3.42-4.83) | Z=833.5 | 0.677 | 0.9 |  |
|  | Yes (n=15) | 4.21±0.5 |  |  |  |  |
| Potential_Humanistic_Care | No (n=119) | 3.67 (2.67-4.33) | Z=888.5 | 0.98 | 0.98 |  |
|  | Yes (n=15) | 3.49±0.8 |  |  |  |  |
| Medical Potential | No (n=119) | 3.78 (3.22-4.67) | Z=842.5 | 0.726 | 0.9 |  |
|  | Yes (n=15) | 3.97±0.57 |  |  |  |  |
| Optimization_Clinical_Efficiency | No (n=119) | 4.17 (3.5-5) | Z=776 | 0.404 | 0.8 |  |
|  | Yes (n=15) | 4.17 (4-5) |  |  |  |  |
| Optimization_Humanistic_Care | No (n=119) | 3.67 (2.67-5) | Z=765.5 | 0.366 | 0.8 |  |
|  | Yes (n=15) | 3.91±0.9 |  |  |  |  |
| Medical Optimization | No (n=119) | 4 (3.22-4.89) | Z=764 | 0.363 | 0.8 |  |
|  | Yes (n=15) | 4.21±0.61 |  |  |  |  |
| Journals | No (n=119) | 0 (0-1) | Z=873 | 0.873 | 0.953 |  |
|  | Yes (n=15) | 0 (0-1) |  |  |  |  |
| Online Courses | No (n=119) | 0 (0-1) | Z=500.5 | < 0.001 | 0.011 |  |
|  | Yes (n=15) | 1 (0.5-1) |  |  |  |  |
| Hospital Training | No (n=119) | 0 (0-0) | Z=671 | 0.037 | 0.186 |  |
|  | Yes (n=15) | 0 (0-1) |  |  |  |  |
| Social Media | No (n=119) | 1 (1-1) | Z=1040 | 0.124 | 0.497 |  |
|  | Yes (n=15) | 1 (0-1) |  |  |  |  |
| Conferences | No (n=119) | 0 (0-0) | Z=908.5 | 0.859 | 0.953 |  |
|  | Yes (n=15) | 0 (0-0) |  |  |  |  |
| Colleague Discussions | No (n=119) | 1 (0-1) | Z=897 | 0.973 | 0.98 |  |
|  | Yes (n=15) | 1 (0-1) |  |  |  |  |
| News & Websites | No (n=119) | 1 (0-1) | Z=963.5 | 0.565 | 0.9 |  |
|  | Yes (n=15) | 0 (0-1) |  |  |  |  |
| Guidelines | No (n=119) | 0 (0-0) | Z=997.5 | 0.164 | 0.612 |  |
|  | Yes (n=15) | 0 (0-0) |  |  |  |  |
| Books | No (n=119) | 0 (0-0) | Z=685.5 | 0.033 | 0.186 |  |
|  | Yes (n=15) | 0 (0-1) |  |  |  |  |
| Vendor Docs | No (n=119) | 0 (0-0) | Z=826.5 | 0.434 | 0.81 |  |
|  | Yes (n=15) | 0 (0-0) |  |  |  |  |
| Other | No (n=119) | 0 (0-0) | Z=915 | 0.545 | 0.9 |  |
|  | Yes (n=15) | 0 (0-0) |  |  |  |  |
| Understanding | No (n=119) | 3 (2-3) | Z=568.5 | 0.016 | 0.111 |  |
|  | Yes (n=15) | 3.53±1.06 |  |  |  |  |
| Reading Articles | No (n=119) | 2 (1-3) | Z=404.5 | < 0.001 | 0.007 |  |
|  | Yes (n=15) | 4 (3-4.5) |  |  |  |  |
| Effective Use | No (n=119) | 3 (2-4) | Z=492.5 | 0.004 | 0.034 |  |
|  | Yes (n=15) | 4 (3-5) |  |  |  |  |
| Access & Use | No (n=119) | 3 (2-4) | Z=501 | 0.005 | 0.038 |  |
|  | Yes (n=15) | 5 (3.5-5) |  |  |  |  |
| Active Seeking | No (n=119) | 3 (3-4) | Z=361 | < 0.001 | 0.005 |  |
|  | Yes (n=15) | 5 (4-5) |  |  |  |  |
| Future Importance | No (n=119) | 4 (3-5) | Z=589 | 0.025 | 0.155 |  |
|  | Yes (n=15) | 5 (4-5) |  |  |  |  |
| Needs_Patient Management | No (n=119) | 4 (3-5) | Z=918 | 0.853 | 0.953 |  |
|  | Yes (n=15) | 4 (3-4.5) |  |  |  |  |
| Needs_Medical History Collection | No (n=119) | 3 (3-4) | Z=726.5 | 0.229 | 0.639 |  |
|  | Yes (n=15) | 4 (3-5) |  |  |  |  |
| Needs_Mental Status Examination | No (n=119) | 3 (2-4) | Z=666 | 0.102 | 0.474 |  |
|  | Yes (n=15) | 4 (3-4) |  |  |  |  |
| Needs_Medical Documentation Writing | No (n=119) | 4 (4-5) | Z=719 | 0.192 | 0.632 |  |
|  | Yes (n=15) | 5 (4-5) |  |  |  |  |
| Needs_Diagnostic Assistance | No (n=119) | 4 (3-5) | Z=856.5 | 0.792 | 0.924 |  |
|  | Yes (n=15) | 4 (4-5) |  |  |  |  |
| Needs_Treatment Planning and Outcome Prediction | No (n=119) | 4 (3.5-5) | Z=909.5 | 0.902 | 0.953 |  |
|  | Yes (n=15) | 4 (3.5-5) |  |  |  |  |
| Needs_Risk Assessment and Prognosis Estimation | No (n=119) | 4 (3-5) | Z=993 | 0.46 | 0.831 |  |
|  | Yes (n=15) | 4 (3-4) |  |  |  |  |
| Needs_Doctor-Patient Communication | No (n=119) | 3 (2-4) | Z=852.5 | 0.775 | 0.923 |  |
|  | Yes (n=15) | 4 (2.5-4) |  |  |  |  |
| Needs_Psychological Interventions | No (n=119) | 3 (2-4) | Z=1060.5 | 0.225 | 0.639 |  |
|  | Yes (n=15) | 3 (2.5-3.5) |  |  |  |  |
| Potential_Patient Management | No (n=119) | 4 (4-5) | Z=976.5 | 0.533 | 0.9 |  |
|  | Yes (n=15) | 4 (3-5) |  |  |  |  |
| Potential_Medical History Collection | No (n=119) | 4 (3-5) | Z=730.5 | 0.235 | 0.639 |  |
|  | Yes (n=15) | 4 (4-5) |  |  |  |  |
| Potential_Mental Status Examination | No (n=119) | 3 (3-5) | Z=831 | 0.658 | 0.9 |  |
|  | Yes (n=15) | 3 (3-4.5) |  |  |  |  |
| Potential_Medical Documentation Writing | No (n=119) | 5 (4-5) | Z=720.5 | 0.182 | 0.632 |  |
|  | Yes (n=15) | 5 (4-5) |  |  |  |  |
| Potential_Diagnostic Assistance | No (n=119) | 4 (3-5) | Z=812.5 | 0.551 | 0.9 |  |
|  | Yes (n=15) | 4 (4-5) |  |  |  |  |
| Potential_Treatment Planning and Outcome Prediction | No (n=119) | 4 (3-5) | Z=847.5 | 0.739 | 0.9 |  |
|  | Yes (n=15) | 4 (4-5) |  |  |  |  |
| Potential_Risk Assessment and Prognosis Estimation | No (n=119) | 4 (4-5) | Z=1019 | 0.342 | 0.8 |  |
|  | Yes (n=15) | 4 (3.5-4.5) |  |  |  |  |
| Potential_Doctor-Patient Communication | No (n=119) | 4 (2-5) | Z=911.5 | 0.893 | 0.953 |  |
|  | Yes (n=15) | 4 (3-4) |  |  |  |  |
| Potential_Psychological Interventions | No (n=119) | 4 (3-5) | Z=959.5 | 0.629 | 0.9 |  |
|  | Yes (n=15) | 4 (3-4) |  |  |  |  |
| Optimization_Patient Management | No (n=119) | 4 (3.5-5) | Z=784.5 | 0.414 | 0.8 |  |
|  | Yes (n=15) | 5 (4-5) |  |  |  |  |
| Optimization_Medical History Collection | No (n=119) | 4 (3-5) | Z=780.5 | 0.407 | 0.8 |  |
|  | Yes (n=15) | 4 (4-5) |  |  |  |  |
| Optimization_Mental Status Examination | No (n=119) | 4 (2-5) | Z=741 | 0.271 | 0.69 |  |
|  | Yes (n=15) | 4 (4-5) |  |  |  |  |
| Optimization_Medical Documentation Writing | No (n=119) | 5 (4-5) | Z=742 | 0.24 | 0.639 |  |
|  | Yes (n=15) | 5 (4-5) |  |  |  |  |
| Optimization_Diagnostic Assistance | No (n=119) | 4 (3-5) | Z=780.5 | 0.403 | 0.8 |  |
|  | Yes (n=15) | 4 (4-5) |  |  |  |  |
| Optimization_Treatment Planning and Outcome Prediction | No (n=119) | 4 (4-5) | Z=838.5 | 0.687 | 0.9 |  |
|  | Yes (n=15) | 4 (4-5) |  |  |  |  |
| Optimization_Risk Assessment and Prognosis Estimation | No (n=119) | 4 (3.5-5) | Z=879 | 0.922 | 0.956 |  |
|  | Yes (n=15) | 4 (4-5) |  |  |  |  |
| Optimization_Doctor-Patient Communication | No (n=119) | 4 (3-5) | Z=841 | 0.709 | 0.9 |  |
|  | Yes (n=15) | 4 (3-4.5) |  |  |  |  |
| Optimization_Psychological Interventions | No (n=119) | 4 (3-5) | Z=842 | 0.715 | 0.9 |  |
|  | Yes (n=15) | 4 (3-4.5) |  |  |  |  |

Table S7：Group differences between Department Director Status

| **Item** | **Group** | **Descriptive Statistics** | **Statistic** | **Raw P-value** | **P-value (FDR)** | **Post-hoc Test** |
| --- | --- | --- | --- | --- | --- | --- |
| AI Exposure & Learning | No (n=120) | 3 (2.33-3.67) | Z=606.5 | 0.089 | 0.355 |  |
|  | Yes (n=14) | 3.52±1.14 |  |  |  |  |
| AI Trust & Expectations | No (n=120) | 3.33 (2.67-4) | Z=603.5 | 0.084 | 0.355 |  |
|  | Yes (n=14) | 3.74±0.87 |  |  |  |  |
| Needs_Clinical_Decision | No (n=120) | 4 (3.25-4.67) | Z=623 | 0.11 | 0.363 |  |
|  | Yes (n=14) | 4.33 (4-5) |  |  |  |  |
| Needs_Routine_Tasks | No (n=120) | 3.75 (3-4.25) | Z=772 | 0.622 | 0.849 |  |
|  | Yes (n=14) | 3.71±0.84 |  |  |  |  |
| Needs_Interaction | No (n=120) | 3.5 (2.5-4) | Z=848.5 | 0.953 | 0.953 |  |
|  | Yes (n=14) | 3.18±1.28 |  |  |  |  |
| Medical Needs | No (n=120) | 3.59±0.85 | t=-1.09 | 0.288 | 0.507 |  |
|  | Yes (n=14) | 3.8±0.68 |  |  |  |  |
| Potential_Clinical_Efficiency | No (n=120) | 4 (3.46-4.83) | Z=689 | 0.27 | 0.507 |  |
|  | Yes (n=14) | 4.58 (3.83-4.83) |  |  |  |  |
| Potential_Humanistic_Care | No (n=120) | 3.67 (2.67-4.33) | Z=778.5 | 0.655 | 0.853 |  |
|  | Yes (n=14) | 3.57±1.19 |  |  |  |  |
| Medical Potential | No (n=120) | 3.83 (3.22-4.56) | Z=713 | 0.356 | 0.587 |  |
|  | Yes (n=14) | 4.1±0.71 |  |  |  |  |
| Optimization_Clinical_Efficiency | No (n=120) | 4 (3.5-5) | Z=635 | 0.129 | 0.38 |  |
|  | Yes (n=14) | 4.75 (4.04-5) |  |  |  |  |
| Optimization_Humanistic_Care | No (n=120) | 3.67 (2.67-5) | Z=766 | 0.588 | 0.845 |  |
|  | Yes (n=14) | 4 (3-4.92) |  |  |  |  |
| Medical Optimization | No (n=120) | 4 (3.33-4.89) | Z=683.5 | 0.253 | 0.506 |  |
|  | Yes (n=14) | 4.56 (3.67-4.92) |  |  |  |  |
| Journals | No (n=120) | 0 (0-1) | Z=1063 | 0.054 | 0.307 |  |
|  | Yes (n=14) | 0 (0-0) |  |  |  |  |
| Online Courses | No (n=120) | 0 (0-1) | Z=492 | 0.002 | 0.118 |  |
|  | Yes (n=14) | 1 (0.25-1) |  |  |  |  |
| Hospital Training | No (n=120) | 0 (0-0) | Z=669 | 0.097 | 0.36 |  |
|  | Yes (n=14) | 0 (0-1) |  |  |  |  |
| Social Media | No (n=120) | 1 (1-1) | Z=799 | 0.662 | 0.853 |  |
|  | Yes (n=14) | 1 (1-1) |  |  |  |  |
| Conferences | No (n=120) | 0 (0-0) | Z=913 | 0.393 | 0.629 |  |
|  | Yes (n=14) | 0 (0-0) |  |  |  |  |
| Colleague Discussions | No (n=120) | 1 (0-1) | Z=804 | 0.76 | 0.869 |  |
|  | Yes (n=14) | 1 (0-1) |  |  |  |  |
| News & Websites | No (n=120) | 1 (0-1) | Z=607 | 0.05 | 0.307 |  |
|  | Yes (n=14) | 1 (1-1) |  |  |  |  |
| Guidelines | No (n=120) | 0 (0-0) | Z=737 | 0.159 | 0.42 |  |
|  | Yes (n=14) | 0 (0-0) |  |  |  |  |
| Books | No (n=120) | 0 (0-0) | Z=888 | 0.614 | 0.849 |  |
|  | Yes (n=14) | 0 (0-0) |  |  |  |  |
| Vendor Docs | No (n=120) | 0 (0-0) | Z=832 | 0.926 | 0.943 |  |
|  | Yes (n=14) | 0 (0-0) |  |  |  |  |
| Other | No (n=120) | 0 (0-0) | Z=861 | 0.561 | 0.826 |  |
|  | Yes (n=14) | 0 (0-0) |  |  |  |  |
| Understanding | No (n=120) | 3 (2-3) | Z=746 | 0.473 | 0.735 |  |
|  | Yes (n=14) | 3 (2.25-3.75) |  |  |  |  |
| Reading Articles | No (n=120) | 2 (1-3) | Z=587.5 | 0.059 | 0.307 |  |
|  | Yes (n=14) | 3 (2.25-3.75) |  |  |  |  |
| Effective Use | No (n=120) | 3 (2-4) | Z=708 | 0.323 | 0.549 |  |
|  | Yes (n=14) | 3.5 (2.25-4.75) |  |  |  |  |
| Access & Use | No (n=120) | 3 (2-4) | Z=656.5 | 0.173 | 0.42 |  |
|  | Yes (n=14) | 3.64±1.34 |  |  |  |  |
| Active Seeking | No (n=120) | 3 (3-4) | Z=667 | 0.194 | 0.452 |  |
|  | Yes (n=14) | 3.5 (3-5) |  |  |  |  |
| Future Importance | No (n=120) | 4 (3-5) | Z=475.5 | 0.006 | 0.154 |  |
|  | Yes (n=14) | 5 (4.25-5) |  |  |  |  |
| Needs_Patient Management | No (n=120) | 4 (3-5) | Z=592 | 0.059 | 0.307 |  |
|  | Yes (n=14) | 4.5 (4-5) |  |  |  |  |
| Needs_Medical History Collection | No (n=120) | 3.5 (3-4) | Z=892 | 0.699 | 0.853 |  |
|  | Yes (n=14) | 3.36±1.01 |  |  |  |  |
| Needs_Mental Status Examination | No (n=120) | 3 (2-4) | Z=894.5 | 0.687 | 0.853 |  |
|  | Yes (n=14) | 2.79±1.42 |  |  |  |  |
| Needs_Medical Documentation Writing | No (n=120) | 4 (4-5) | Z=685 | 0.23 | 0.495 |  |
|  | Yes (n=14) | 5 (4-5) |  |  |  |  |
| Needs_Diagnostic Assistance | No (n=120) | 4 (3-5) | Z=599.5 | 0.066 | 0.307 |  |
|  | Yes (n=14) | 4.5 (4-5) |  |  |  |  |
| Needs_Treatment Planning and Outcome Prediction | No (n=120) | 4 (3-5) | Z=661 | 0.169 | 0.42 |  |
|  | Yes (n=14) | 4.5 (4-5) |  |  |  |  |
| Needs_Risk Assessment and Prognosis Estimation | No (n=120) | 4 (3-5) | Z=700.5 | 0.29 | 0.507 |  |
|  | Yes (n=14) | 4 (4-5) |  |  |  |  |
| Needs_Doctor-Patient Communication | No (n=120) | 3 (2-4) | Z=857 | 0.902 | 0.939 |  |
|  | Yes (n=14) | 3.14±1.35 |  |  |  |  |
| Needs_Psychological Interventions | No (n=120) | 3 (2-4) | Z=861 | 0.878 | 0.939 |  |
|  | Yes (n=14) | 3.21±1.37 |  |  |  |  |
| Potential_Patient Management | No (n=120) | 4 (3-5) | Z=631 | 0.109 | 0.363 |  |
|  | Yes (n=14) | 5 (4-5) |  |  |  |  |
| Potential_Medical History Collection | No (n=120) | 4 (3-5) | Z=872 | 0.811 | 0.891 |  |
|  | Yes (n=14) | 3.71±1.27 |  |  |  |  |
| Potential_Mental Status Examination | No (n=120) | 3 (3-5) | Z=929.5 | 0.505 | 0.765 |  |
|  | Yes (n=14) | 3.5 (2.25-4) |  |  |  |  |
| Potential_Medical Documentation Writing | No (n=120) | 5 (4-5) | Z=646 | 0.121 | 0.377 |  |
|  | Yes (n=14) | 5 (4.25-5) |  |  |  |  |
| Potential_Diagnostic Assistance | No (n=120) | 4 (3-5) | Z=597 | 0.061 | 0.307 |  |
|  | Yes (n=14) | 5 (4-5) |  |  |  |  |
| Potential_Treatment Planning and Outcome Prediction | No (n=120) | 4 (3-5) | Z=687 | 0.24 | 0.497 |  |
|  | Yes (n=14) | 5 (4-5) |  |  |  |  |
| Potential_Risk Assessment and Prognosis Estimation | No (n=120) | 4 (4-5) | Z=698 | 0.272 | 0.507 |  |
|  | Yes (n=14) | 4.5 (4-5) |  |  |  |  |
| Potential_Doctor-Patient Communication | No (n=120) | 3 (2-5) | Z=586.5 | 0.059 | 0.307 |  |
|  | Yes (n=14) | 4.5 (3.25-5) |  |  |  |  |
| Potential_Psychological Interventions | No (n=120) | 4 (3-5) | Z=803 | 0.784 | 0.878 |  |
|  | Yes (n=14) | 4 (2.25-4.75) |  |  |  |  |
| Optimization_Patient Management | No (n=120) | 4 (3-5) | Z=686 | 0.23 | 0.495 |  |
|  | Yes (n=14) | 5 (4-5) |  |  |  |  |
| Optimization_Medical History Collection | No (n=120) | 4 (3-5) | Z=824 | 0.905 | 0.939 |  |
|  | Yes (n=14) | 4 (4-5) |  |  |  |  |
| Optimization_Mental Status Examination | No (n=120) | 4 (3-5) | Z=798.5 | 0.758 | 0.869 |  |
|  | Yes (n=14) | 4 (2.5-5) |  |  |  |  |
| Optimization_Medical Documentation Writing | No (n=120) | 5 (4-5) | Z=665 | 0.159 | 0.42 |  |
|  | Yes (n=14) | 5 (4.25-5) |  |  |  |  |
| Optimization_Diagnostic Assistance | No (n=120) | 4 (3-5) | Z=555.5 | 0.028 | 0.307 |  |
|  | Yes (n=14) | 5 (4-5) |  |  |  |  |
| Optimization_Treatment Planning and Outcome Prediction | No (n=120) | 4 (3-5) | Z=526 | 0.015 | 0.276 |  |
|  | Yes (n=14) | 5 (4.25-5) |  |  |  |  |
| Optimization_Risk Assessment and Prognosis Estimation | No (n=120) | 4 (3-5) | Z=577 | 0.04 | 0.307 |  |
|  | Yes (n=14) | 5 (4.25-5) |  |  |  |  |
| Optimization_Doctor-Patient Communication | No (n=120) | 4 (3-5) | Z=796 | 0.743 | 0.869 |  |
|  | Yes (n=14) | 4 (3.25-5) |  |  |  |  |
| Optimization_Psychological Interventions | No (n=120) | 4 (3-5) | Z=788.5 | 0.701 | 0.853 |  |
|  | Yes (n=14) | 4 (3.25-5) |  |  |  |  |
